# *Ortho*-Hydroxyanilides: Slow-Acting, Selective Histone Deacetylase 1/2 Inhibitors Suitable for Photocaging Applications

**DOI:** 10.1021/acsptsci.5c00562

**Published:** 2025-11-14

**Authors:** Irina Honin, Tao Sun, Nisha Setia, Linda Schäker-Hübner, Finn K. Hansen

**Affiliations:** Department of Pharmaceutical and Cell Biological Chemistry, Pharmaceutical Institute, 9374 University of Bonn, An der Immenburg 4, 53121 Bonn, Germany

**Keywords:** histone deacetylase (HDAC), HDAC inhibitors (HDACi), cancer, epigenetics, photocaging, light-activatable drugs

## Abstract

Histone deacetylases (HDACs) regulate gene expression and are promising targets in oncology. Especially the class I isoforms HDAC1 and HDAC2 are overexpressed in cancer. However, while *ortho*-aminoanilides with a suitable (het)­aryl substitution are well-characterized HDAC1/HDAC2 inhibitors, the corresponding phenol analogs have not been sufficiently investigated so far. To this end, we compared the *ortho*-hydroxyanilide derivative **ST13** with the pan-HDAC inhibitor vorinostat and Cpd-60, an *ortho*-aminoanilide with high HDAC1/HDAC2 selectivity. **ST13** was further developed into a light-activatable prodrug (**ST17**) by masking its zinc-binding group with a photoremovable 4,5-dimethoxy-2-nitrobenzyl protecting group. Overall, we verified that **ST13** is a selective, slow- and tight-binding HDAC1/HDAC2 inhibitor with antiproliferative activity. Furthermore, we demonstrated that the light-activatable prodrug **ST17** readily releases **ST13** upon irradiation, thereby allowing to precisely control its antiproliferative properties. These findings validate *ortho*-hydroxyanilides as effective HDAC1/HDAC2-selective inhibitors and highlight photocaging as a promising strategy to achieve spatiotemporal control of epigenetic therapies in cancer.

## Graphical abstract

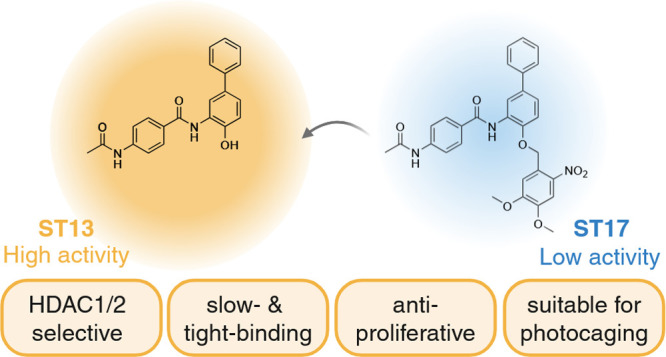

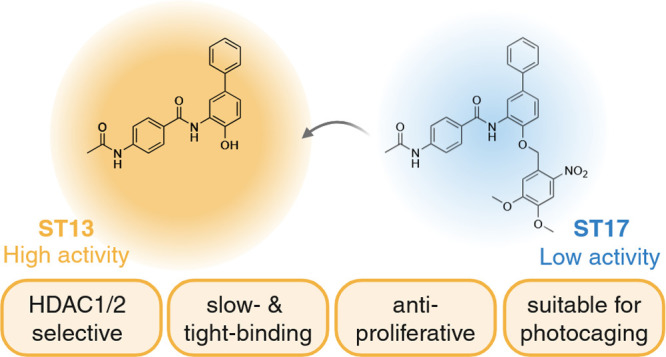

Histone deacetylases (HDACs) play a crucial role in the remodeling of the chromatin structure and regulating gene expression.^1,2^ Alongside histone acetyltransferases (HATs), they modulate chromatin structures by regulating histone acetylation at lysine residues. HDACs catalyze the removal of acyl groups from lysines, leading to chromatin compaction and transcriptional repression, whereas HATs promote chromatin relaxation and transcriptional activation.^1,2^ Mammalian HDACs are classified into two primary groups based on their homology with yeast deacetylases and their mechanism of action. The first group is zinc-dependent and comprises four classes: class I (HDAC1, HDAC2, HDAC3, and HDAC8), class IIa (HDAC4, HDAC5, HDAC7, and HDAC9), class IIb (HDAC6 and HDAC10), and class IV (HDAC11). The second group contains class III HDACs, also known as sirtuins (SIRT1–7), which require nicotinamide adenine dinucleotide (NAD^+^) as a cosubstrate.^1,2^ However, HDACs also deacetylate a myriad of nonhistone proteins, including transcription factors (e.g., p53 ^3^ and STAT3 ^4^), chaperones (e.g., Hsp90 ^5^), cytoskeletal proteins (e.g., α-tubulin^6^), and cell cycle regulators (e.g., cyclin-dependent kinases^7^). Thus, HDACs are involved in various biological processes and their dysregulated expression has been observed in several diseases such as cancer, neurological disorders, infections, and inflammatory conditions.^8^

To date, regulatory agencies have approved six HDAC inhibitors. The Food and Drug Administration (FDA) has approved vorinostat, belinostat, romidepsin, and panobinostat for the treatment of malignant hematological diseases such as T-cell lymphoma and multiple myeloma.^9^ Furthermore, tucidinostat (chidamide) has been approved by the Chinese National Medical Products Administration (NMPA) for the treatment of peripheral T-cell lymphoma and advanced breast cancer, making it the first and only HDAC inhibitor approved for the treatment of a solid cancer.^10^ Moreover, the recent approval of givinostat for *Duchenne* muscular dystrophy (DMD) highlights the potential of HDAC inhibitors in nononcological diseases.^11,12^ However, the lack of selectivity of currently approved HDAC inhibitors limits their therapeutic application, as their use is associated with severe adverse side effects, such as gastric issues, hematologic imbalances, or cardiac disturbances.^8^ Therefore, it is important to develop potent and selective inhibitors to avoid such severe, dose-limiting side effects.^13,14^

In this context, HDAC1/HDAC2 selective inhibitors are especially promising drug candidates, particularly in the field of oncology: Aberrant expression of these isoforms has been associated with various diseases, e.g., breast, prostate, lung, stomach, colon, and skin cancer as well as hematopoietic malignancies.^1,15–17^ Selective targeting of HDAC1/HDAC2 can be achieved through subtle modifications of the HDAC inhibitor pharmacophore model (see Figure 1) which is typically composed of a zinc-binding group (ZBG), which coordinates the zinc ion in the active site, a cap group, which interacts with the surface of the enzyme, and a linker that connects both elements. HDAC1/HDAC2 selective HDAC inhibitors can be effectively designed by selecting an appropriate ZBG. The widely used hydroxamic acid ZBG, as utilized in vorinostat (Figure 1A), often lacks isoform selectivity.^18^ In contrast, alternative ZBGs like *ortho*-aminoanilides (Figure 1B) or alkyl hydrazides (Figure 1C) have demonstrated increased potency and selectivity toward HDAC1–3.^19–22^ In particular, *ortho*-aminoanilides are often considered the gold standard for class I selective HDAC inhibitors and have been extensively studied.^22–24^ Furthermore, HDAC1/HDAC2 selectivity can be achieved by targeting the so-called foot pocket, which is a lipophilic internal cavity specific for class I HDACs.^25^ Typically, aromatic moieties such as phenyl, thienyl, or pyridinyl are employed as foot pocket units (FPUs) to engage the foot pocket of HDAC1/HDAC2.^20^ More recently, hybrid design strategies that combine the hydroxamic acid ZBG with additional pharmacophoric elements have been reported to improve the selectivity of hydroxamic acid based inhibitors.^18^

**Figure 1:**
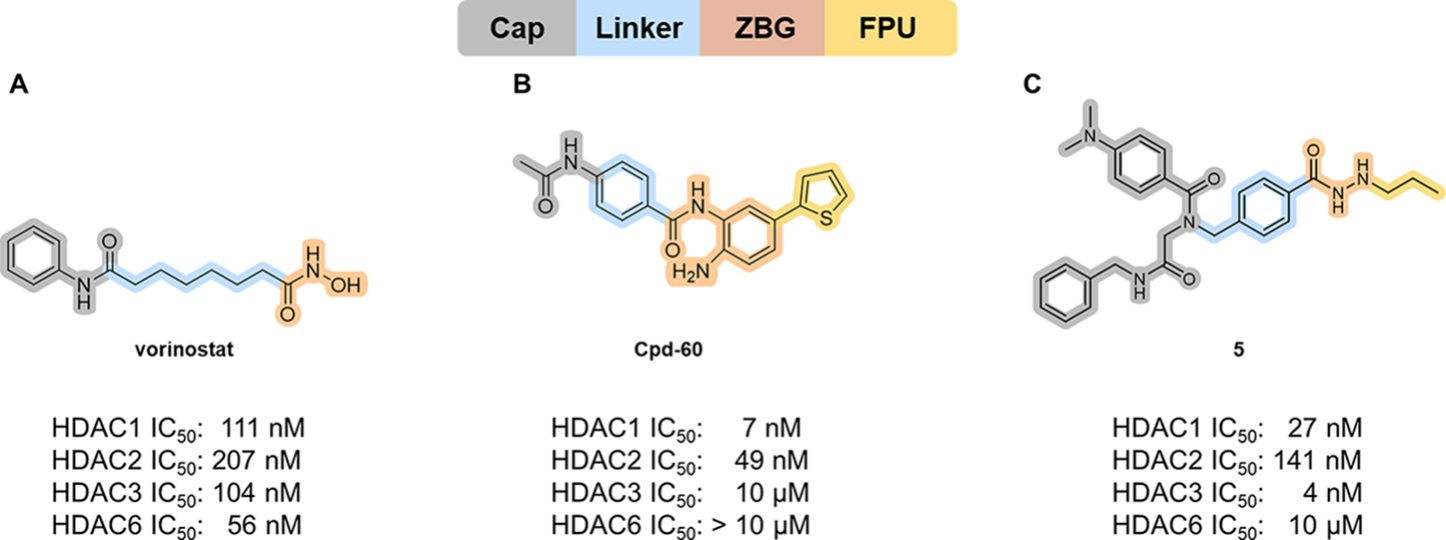
Selected HDAC inhibitors with pharmacophore model and IC_50_ values for HDAC1–3 and HDAC6: The FDA-approved HDAC inhibitor vorinostat (A), the HDAC1/HDAC2 selective inhibitor Cpd-60 with an *ortho*-aminoanilide ZBG (B), and compound **5** featuring a propyl hydrazide as ZBG (C). Data taken from refs 19 and 24 .

Another attractive approach to overcome the unwanted cytotoxicity of conventional HDAC inhibitors involves photopharmacological strategies, which enable precise spatial and temporal control over drug activity.^26,27^ Ideal light-activatable inhibitors are inactive in their caged form and only become biologically active upon exposure to light of a specific wavelength. This approach minimizes toxicity outside the disease location and allows for localized activation in the targeted tissues, making it particularly interesting for the treatment of solid tumors.^28^ Photocaged inhibitors possess photoremovable protecting groups, such as nitroaryl or coumarin-4-ylmethyl groups, which mask essential pharmacophoric features.^27^ Upon irradiation with light of a certain wavelength, the photolabile group is cleaved, thereby restoring the inhibitor’s potency.^27^ Light-activatable prodrugs have already been developed for a variety of anticancer drugs, e.g., vermurafenib, paclitaxel, and doxorubicin.^29–33^ In the epigenetic field, photoprotected derivatives of vorinostat (AC-SAHA,^34^ p-Fc-SAHA^35^), panobinostat (zap-Pano^36^), and DDK137 (pc-I^26^) have been developed. However, all photocaged HDAC inhibitors described so far are based on nonselective HDAC inhibitors.

In this work, we present the synthesis and in-depth characterization of **ST13**, a selective HDAC1/HDAC2 inhibitor that features an *ortho*-hydroxyanilide ZBG bearing a phenyl ring as FPU (see Figure 2). Overall, this moiety is a relatively unexplored structural feature of selective HDAC1/HDAC2 inhibitors. Therefore, our aim was to compare **ST13** with Cpd-60 (see Figure 2), a well-investigated HDAD1/HDAC2 selective *ortho*-aminoanilide HDAC inhibitor. To this end, both compounds were thoroughly characterized concerning their inhibition of selected HDAC isoforms, binding kinetics as well as their antiproliferative effects against breast and skin cancer cell lines. This approach allowed us to gain deeper insights into their selectivity profiles, binding modes, and functional properties, providing a basis for further optimization of class I selective HDAC inhibitors. In addition, we developed **ST17** (see Scheme 1), a photocaged HDAC1/HDAC2 selective inhibitor incorporating a 4,5-dimethoxy-2-nitrobenzyl (DMNB) moiety as photoremovable protecting group.

**Figure 2:**
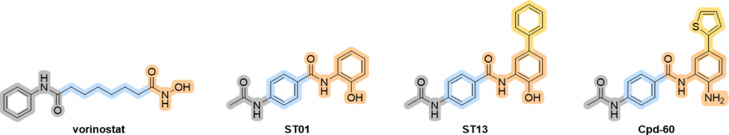
Structures of tested compounds (**ST13**, Cpd-60) and controls (vorinostat, **ST01**).

**Scheme 1:**
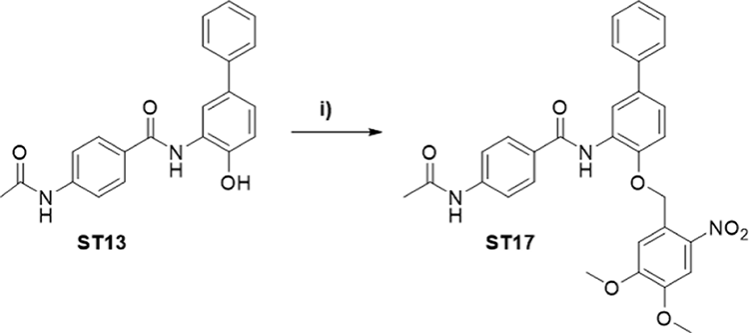
Synthesis of Photocaged HDAC Inhibitor **ST17** ^a^

a Reagents and conditions: (i) 1-(bromomethyl)-4,5-dimethoxy-2-nitrobenzene, K_2_CO_3_, acetone, 60 °C, 12 h, 43% yield.

## Results and Discussion

In contrast to *ortho*-aminoanilides, HDAC inhibitors with an *ortho*-hydroxyanilides scaffold have received little attention to date. Investigations of *ortho*-hydroxyanilides as HDAC inhibitors have so far been limited to the studies of Methot *et al.* ^24^ Furthermore, Jaikhan et al. studied *ortho*-hydroxyanilide-based derivatives as lysine demethylase inhibitors but observed only very weak HDAC activity.^37^ To investigate the functional differences between *ortho*-hydroxyanilides and *ortho*-aminoanilides as selective HDAC1/HDAC2 inhibitors, we focused on representative compounds from each class. The *ortho*-hydroxyanilide derivative **ST13** was chosen based on its high inhibitory potency reported by Methot *et al.* ^24^ As shown in Figure 2, **ST13** features a phenyl group as a FPU and was previously described with an IC_50_ value of 0.018 μM for HDAC1. **ST01**, a FPU-free analog of **ST13**, was included to evaluate the impact of the FPU unit (see the Supporting Information for synthetic details). For the *ortho*-aminoanilide class, Cpd-60 was selected due to its strong potency and selectivity for HDAC1 (IC_50_ = 0.007 μM) and HDAC2 (IC_50_ = 0.049 μM), with markedly reduced activity against other HDAC isoforms (HDAC3: IC_50_ = 10 μM; HDAC4–8: IC_50_ > 10 μM).^24^

### *In Vitro* HDAC Inhibition Assays

First, the inhibitory activity and isoform selectivity of compounds and controls (see Figure 2) was evaluated in a fluorescence-based biochemical HDAC inhibition assay against recombinant HDAC1, HDAC2, HDAC3, and HDAC6. As expected, **ST13** showed potent inhibitory activity against HDAC1 (IC_50_ = 0.023 μM) and HDAC2 (IC_50_ = 0.049 μM) as well as weak inhibition of HDAC3 (IC_50_ = 4.30 μM) and HDAC6 (IC_50_ > 10 μM). In our assay-setting, Cpd-60 exhibited similar IC_50_ values as **ST13** (HDAC1: IC_50_ = 0.008 μM; HDAC2: IC_50_ = 0.039 μM) which are also in good agreement with literature.^24^ In contrast, **ST01**, the FPU-free equivalent of **ST13**, showed no activity against HDAC1 and HDAC2 up to 10 μM and was therefore excluded from further investigation (Table 1).

**Table 1:** Inhibitory Activity of **ST13**, **ST01**, Cpd-60 and Vorinostat against HDAC1, HDAC2, HDAC3 and HDAC6

	IC_50_ [μM]			
	HDAC1^a^ ^,^ ^b^	HDAC2^a^ ^,^ ^b^	HDAC3^a^ ^,^ ^b^	HDAC6^a^ ^,^ ^c^
**ST13**	0.023 ± 0.004	0.049 ± 0.003	4.30 ± 1.12	>10^d^
**ST01**	>10^d^	>10^d^	n.d.	n.d.
Cpd-60	0.008 ± 0.001	0.039 ± 0.007	1.42 ± 0.04 ^20^	>10 ^20^
vorinostat	0.111 ± 0.009	0.207 ± 0.021	0.104 ± 0.017	0.056 ± 0.008

a IC_50_ values are reported as mean ± standard deviation (SD) from at least two independent experiments.

b Preincubation of enzyme and inhibitor: 1 h at RT.

c Preincubation of enzyme and inhibitor: 15 min at RT.

d <30% inhibition at the stated concentration; n.d.: not determined.

### Preincubation Studies

Subsequently, we investigated the influence of the preincubation time on the IC_50_ values for HDAC1 and HDAC2 to gain insights into the association behavior.^38^ Therefore, HDAC1 and HDAC2 were treated with **ST13**, Cpd-60, and vorinostat for varying times of preincubation (Table 2). As expected, in the case of vorinostat, the IC_50_ values of HDAC1 and HDAC2 remained unaffected by the preincubation time, confirming its fast-binding mechanism.^39–41^ For both **ST13** and Cpd-60 we observed a pronounced dependency between preincubation time and the respective IC_50_ values, suggesting that, similar to *ortho*-aminoanilides, *ortho*-hydroxyanilide derivatives act as slow-binding HDAC1/HDAC2 inhibitors.

**Table 2:** Analysis of the Effect of Preincubation Time on **ST13**, Cpd-60, and Vorinostat at HDAC1 and HDAC2

	HDAC1 IC_50_ [μM]^a^				HDAC2 IC_50_ [μM]^a^			
preincubationtime	5 min	30 min	60 min	120 min	5 min	30 min	60 min	120 min
**ST13**	0.055 ± 0.007	0.036 ± 0.004	0.034 ± 0.005	0.025 ± 0.003	0.082 ± 0.012	0.083 ± 0.003	0.075 ± 0.005	0.049 ± 0.009
Cpd-60	0.018 ± 0.003	0.011 ± 0.002	0.008 ± 0.001	0.005 ± 0.001	0.057 ± 0.006	0.052 ± 0.005	0.039 ± 0.007	0.038 ± 0.003
vorinostat	0.118 ± 0.030	0.123 ± 0.032	0.121 ± 0.012	0.119 ± 0.015	0.207 ± 0.037	0.232 ± 0.018	0.201 ± 0.009	0.228 ± 0.003

a IC_50_ values are reported as mean ± SD from at least two independent experiments.

### Jump Dilution Assays

Next, the dissociation behavior of **ST13** and Cpd-60 was evaluated in 100-fold jump dilution experiments using vorinostat as a control. The assay was performed according to a previously published protocol with minor modifications.^20^ Briefly, the HDAC1 and HDAC2 enzymes were incubated for 1 h with a concentration of the test compounds corresponding to the 5-fold (HDAC1) or 10-fold (HDAC2) of the previously determined IC_50_ values (“incubation mix”). Subsequently, this “incubation mix” was diluted 1:100, and the dissociation of test compounds was monitored continuously. As expected, vorinostat, known as a fast-on/fast-off binding HDAC inhibitor, rapidly dissociated from HDAC1 and HDAC2 upon 100-fold dilution. In contrast, **ST13** and Cpd-60 exhibited tight-binding properties to HDAC1 and HDAC2 (Figure 3).

**Figure 3:**
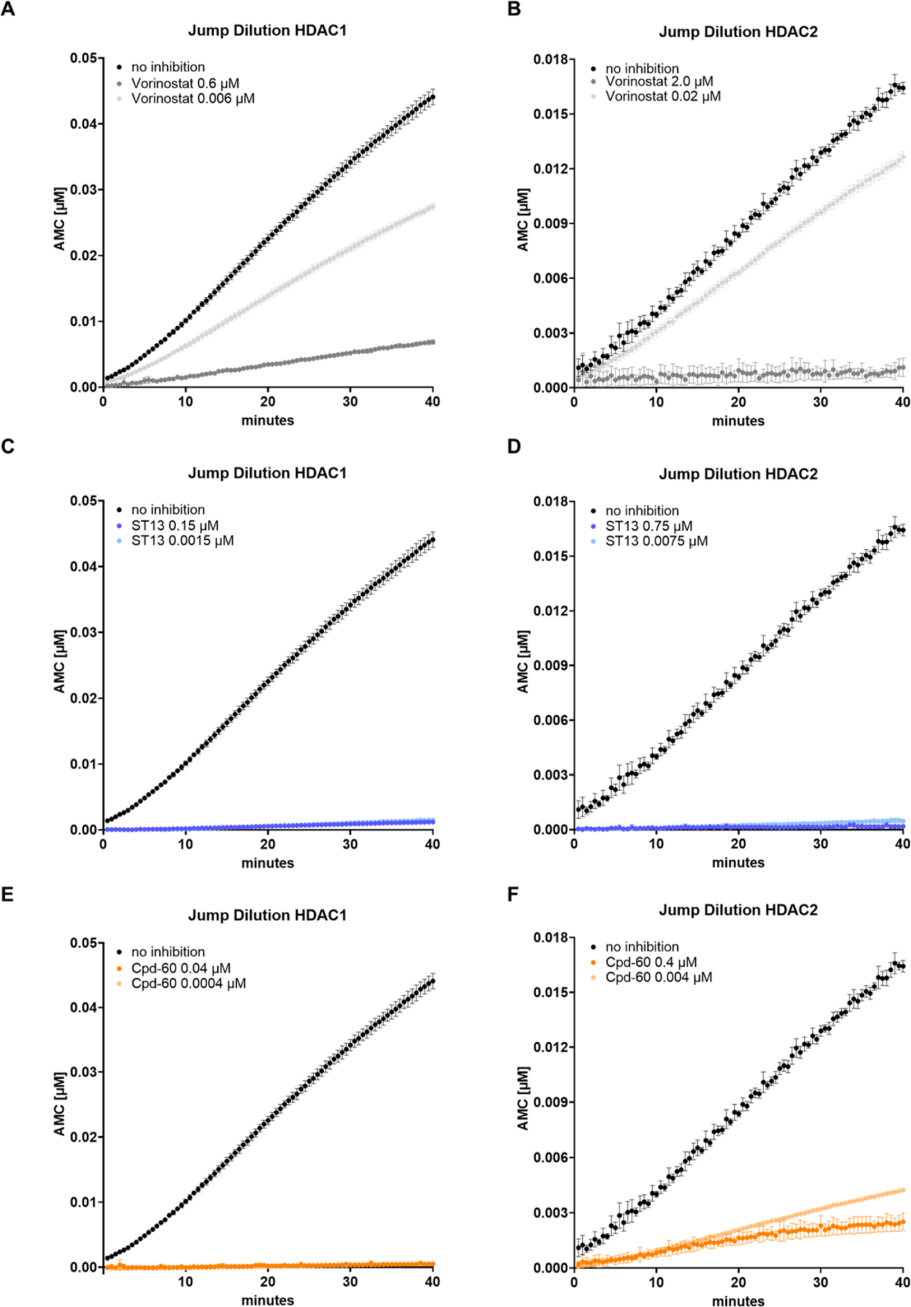
Progression curves of 100-fold jump dilution experiments of vorinostat (A,B), **ST13** (C,D) and Cpd-60 (E,F) at HDAC1 (left) and HDAC2 (right) to study their dissociation behavior. The isoforms were incubated with concentrations of the respective inhibitors corresponding to either 5-fold (HDAC1) or 10-fold (HDAC2) of their IC_50_ values. The specific concentrations are indicated in the respective graphs. The enzymes were preincubated with the inhibitor for 1 h before 1:100 dilution. Representative curves of 100-fold jump dilution experiments at HDAC1 and HDAC2 are shown. The assay was performed in triplicates and repeated at least twice.

### Determination of the Binding Mechanism

Furthermore, we assessed the slow-binding mechanism of **ST13** and Cpd-60. The two most common mechanisms of slow-binding inhibition are “simple slow-binding” (mechanism I) and “induced fit” (mechanism II).^38^ As illustrated in Figure 4, the simple slow-binding mechanism is a single-step mechanism with a slow association and dissociation rate. In contrast, the induced fit mechanism proceeds via a two-step process: in the first step the enzyme and the inhibitor rapidly form an encounter complex (EI), which subsequently converts slowly to a stable complex E*I. The latter mechanism is observed more commonly for slow-binding inhibitors.^38^

**Figure 4:**
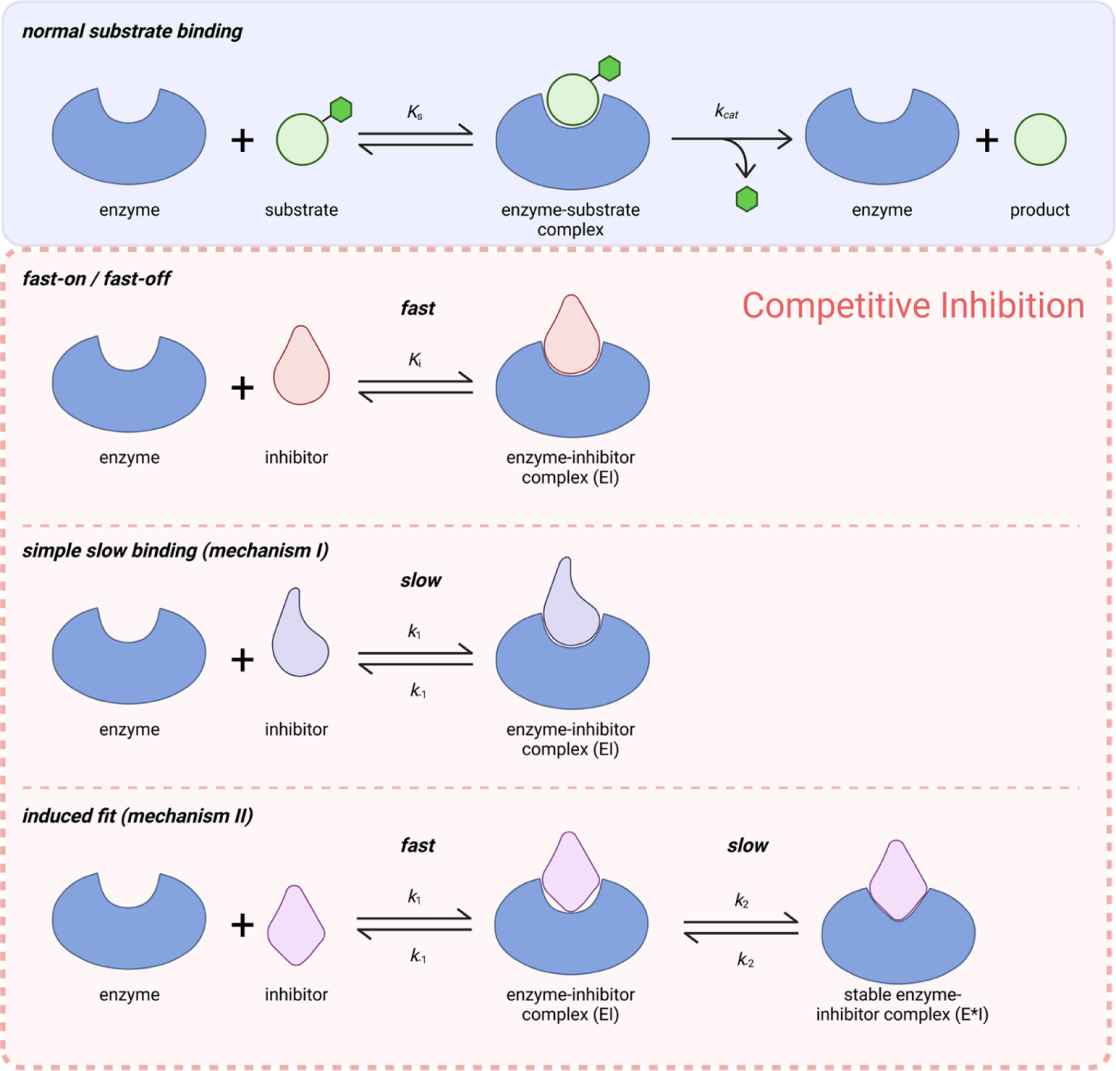
Representative examples of different kinetic mechanisms of enzyme inhibition. Simple slow-binding mechanism is a single-step mechanism with slow rate constants (*k*_1_/*k*_–1_). Inhibitors following an induced fit mechanism form an encounter complex of modest affinity fast (EI), which subsequently slowly isomerizes to a complex with higher affinity (E*I).

To determine the slow-binding mechanism of **ST13** and Cpd-60, the HDAC assay was conducted in a continuous format. To this end, HDAC1, the substrate, and trypsin were incubated directly with the inhibitor at various concentrations and fluorescence was recorded continuously for 45 min. Vorinostat was previously described as a fast-on/fast-off inhibitor and employed as an assay control. It rapidly reached enzyme–inhibitor equilibrium and exhibited linear progression curves at all inhibitor concentrations (Figure S1A, Supporting Information). By plotting the ratio of the deacetylation rate in the presence of the inhibitor (*v*_i_) to the rate in absence of any inhibitor (*v*_0_) as a function of the inhibitor concentration and fitting the data to an appropriate model (eq 3), the equilibrium rate constant *K*_i_ was determined (Figure 5). For vorinostat a *K*_i_ of 0.041 ± 0.015 μM for HDAC1 was obtained, which is in agreement with previously published data.^20^

**Figure 5:**
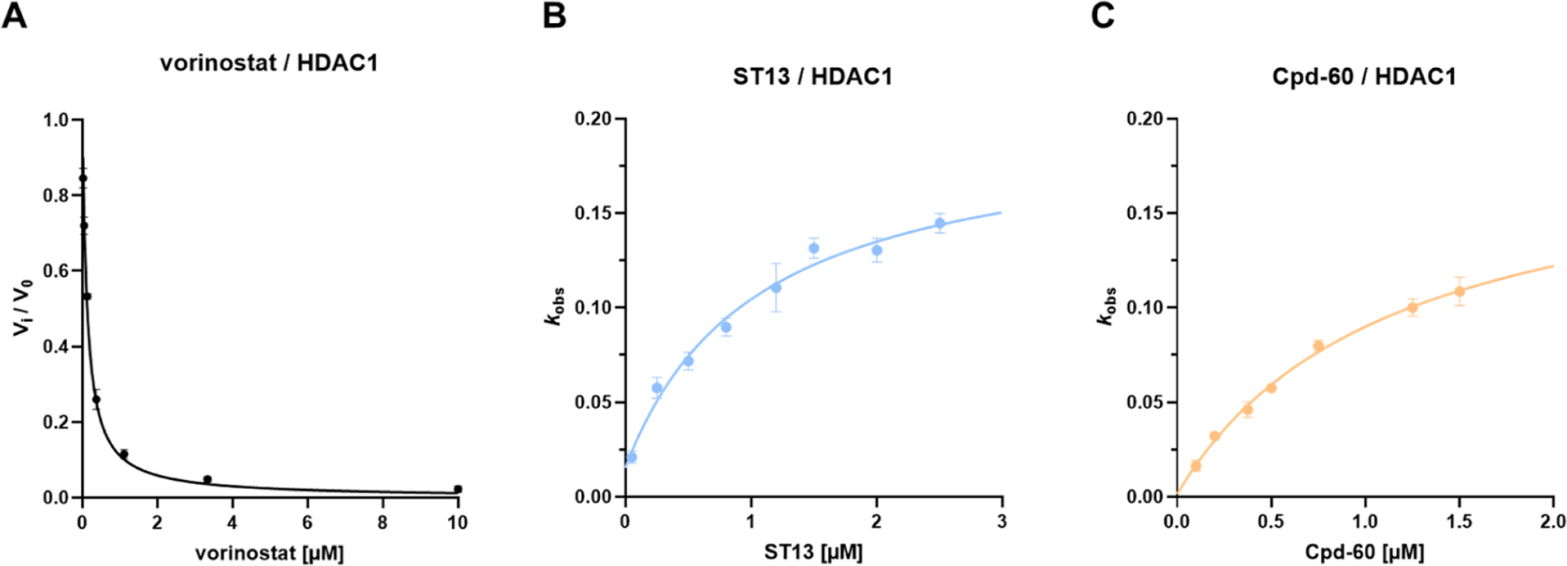
Kinetics of binding of **ST13**, Cpd-60, and vorinostat to HDAC1. (A) For vorinostat the ratio of the steady-state velocities *v*_i_/*v*_0_ was plotted against the corresponding inhibitor concentrations to determine the *K*_i_ value and fitted to eq 3. (B,C) The apparent first-order rate constants for equilibrium (*k*_obs_) versus the corresponding inhibitor concentration was fitted to eq 4 (mechanism II of slow-binding kinetics). Representative curves are shown. The assay was performed in triplicates and repeated at least twice.

In contrast, slow-binding inhibitors like **ST13** and Cpd-60 reduced the substrate conversion rate over time, resulting in “bent” progression curves (Figure S1B, Supporting Information) with two linear phases.^38^ The first phase reflects the initial velocity, while the second represents the steady-state velocity after the enzyme–inhibitor equilibrium had been reached.^38^ The rate of approach to equilibrium, referred to as the observed rate constant (*k*_obs_), describes the transition between these two phases and can be used to distinguish between mechanisms I and II: Plotting *k*_obs_ values as a function of the inhibitor concentration reveals whether the inhibitor follows mechanism I (linear plot) or mechanism II (hyperbolic plot) of slow-binding inhibition.^38^ As shown in Figure 5, for both inhibitors *k*_obs_ increased hyperbolically with the inhibitor concentration, indicating that **ST13** and Cpd-60 inhibit HDAC1 via the induced fit slow-binding mechanism. To investigate the binding mode of both compounds, we performed molecular docking studies. Specifically, **ST13** was docked into HDAC2 (PDB ID: 3MAX), a cocrystal structure containing an HDAC inhibitor with an *ortho*-aminoanilide ZBG and a phenyl group as the FPU. Comparison of the docking pose of **ST13** with that of the structurally related *ortho*-aminoanilide showed that, despite their distinct ZBGs, both ligands adopt similar orientations within the HDAC2 binding site (see Figure S5, Supporting Information). These results suggest that the *ortho*-hydroxyanilide ZBG of **ST13**, together with its phenyl FPU, serves as a bioisosteric replacement for the *ortho*-aminoanilide ZBG in Cpd-60, which may also account for the observed kinetic similarities between **ST13** and Cpd-60.

### Inhibitory HDAC Activity in a Cellular Environment

Next, we examined the effects of our slow- and tight-binding HDAC inhibitors in a cellular environment using an HDAC whole cell assay. To this end, melanoma (MV-3) and triple-negative breast (MDA-MB-231) cancer cells were treated with **ST13** and Cpd-60. As shown in Table 3, both compounds exhibited an inhibitory effect against HDACs after 18 h of incubation. In MV-3 cells, **ST13** (IC_50_ = 0.311 μM) and Cpd-60 (IC_50_ = 0.097 μM) displayed more potent inhibitory activity compared to vorinostat (IC_50_ = 0.406 μM). In MDA-MB-231 cells Cpd-60 (IC_50_ = 0.087 μM) showed superior efficacy over vorinostat (IC_50_ = 0.240 μM) and **ST13** (IC_50_ = 0.577 μM). Furthermore, we determined the EC_50_ values in the nontumorigenic cell line HEK293 and in the hematological cancer cell line MM.1S. In both cell lines, we observed similar EC_50_ values like in MV-3 and MDA-MB-231 cells.

**Table 3:** Inhibitory Activity of **ST13**, Cpd-60, and Vorinostat in HDAC Whole Cell Assays Conducted in Selected Cell Lines

	EC_50_ [μM]^a^			
	MV-3	MDA-MB-231	HEK293	MM.1S
**ST13**	0.311 ± 0.047	0.577 ± 0.097	0.333 ± 0.064	0.637 ± 0.158
Cpd-60	0.097 ± 0.026	0.087 ± 0.008	0.178 ± 0.023	0.111 ± 0.038
vorinostat	0.406 ± 0.069	0.240 ± 0.052	0.405 ± 0.092	0.388 ± 0.031

a EC_50_ values are reported as mean ± SD from at least two independent experiments.

### Association and Dissociation Behavior in a Cellular Environment

The impact on the acetylation levels of the HDAC substrates Ac-histone H3 (class I HDACs) and Ac-α-tubulin (HDAC6) was analyzed by immunoblot analysis. As expected, **ST13** and Cpd-60 only led to hyperacetylation of histone H3, indicating selectivity toward class I HDACs and the absence of HDAC6 inhibition in a cellular environment. Interestingly, a substantial increase in acetylated histone H3 was observed after 72 h of incubation with **ST13** and Cpd-60. Furthermore, immunoblot analysis revealed persistent histone H3 acetylation for Cpd-60 and **ST13** treated cells up to 6 h after wash out, suggesting a low dissociation rate in cells. These findings are in good agreement with the results of the cell-free biochemical assays (e.g., preincubation studies and jump dilution experiments) and provide further evidence that Cpd-60 and **ST13** are slow- and tight-binding HDAC1/HDAC2 inhibitors (Figure 6).

**Figure 6:**
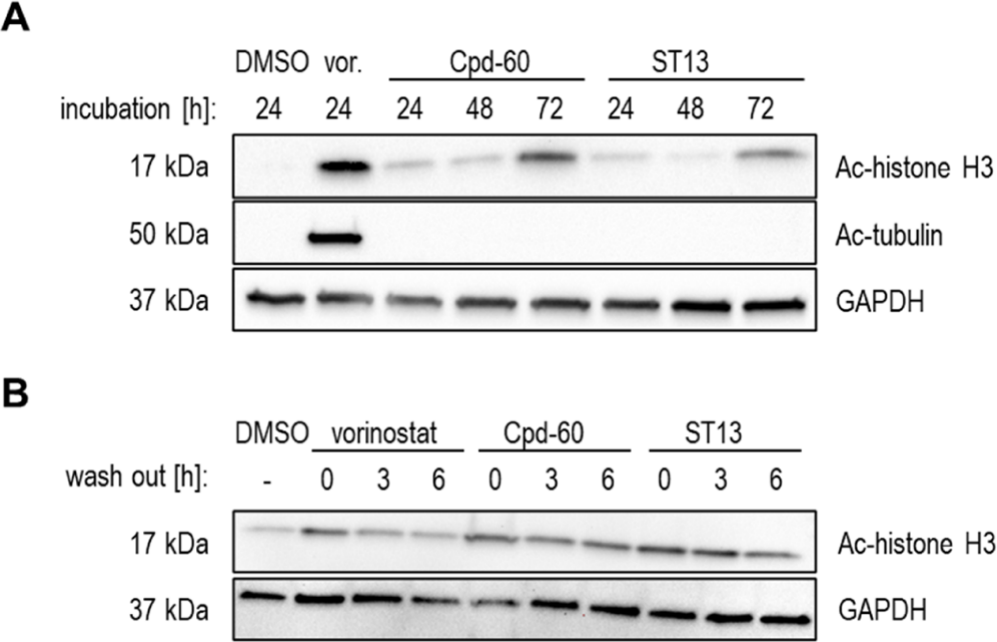
Immunoblot analysis of MV-3 cell lysates. (A) Protein levels of acetylated histone H3 and α-tubulin after treatment with vehicle (DMSO) or 5 μM of vorinostat (vor.), Cpd-60, or **ST13** for 24–72 h, respectively. (B) Protein levels of acetylated histone H3 after wash out experiments: MV-3 lysates following 48 h of incubation with vehicle (DMSO) or 5 μM vorinostat, Cpd-60, or **ST13**, respectively were washed and further incubated without inhibitor for 0 h, 3 h or 6 h in FBS-free cell culture medium. Representative images from at least three replicates are shown. DMSO (0.025%) was used as vehicle control and GAPDH as loading control.

### Influence on Cell Viability

To evaluate the cytotoxicity of the selected HDAC inhibitors, compounds were tested for their antiproliferative activity against MV-3 and MDA-MB-231 cells using the MTT assay. The results are summarized in Table 4. Interestingly, we observed notable time-dependent antiproliferative effects of **ST13** and Cpd-60. In MV-3 cells, the EC_50_ values for **ST13** and Cpd-60 decreased from initial weak antiproliferative activity (<25% viability reduction at 15 μM) after 72 h of incubation to 2.77 μM (**ST13**) and 0.37 μM (Cpd-60) after 120 h of incubation. In contrast, the EC_50_ values for the fast-on/fast-off HDAC inhibitor vorinostat did not shift substantially (1.88 μM after 72 h vs 1.23 μM after 120 h). The same trend was observed in MDA-MB-231 cells. Based on these observations, we extended our investigations and additionally determined EC_50_ values in HEK293 cells and MM.1S cells (see Supporting Information, Table S1). After 120 h of incubation, **ST13** (EC_50_ = 0.198 μM) and Cpd-60 (EC_50_ = 0.111 μM) showed more potent antiproliferative effects compared to vorinostat (EC_50_ = 0.388 μM) in MM.1S cells. Notably, in the nontumorigenic HEK293 cell line (incubation time 120 h), **ST13** showed no relevant toxicity up to a concentration of 5 μM, whereas vorinostat and Cpd-60 showed moderate antiproliferative activity (vorinostat: EC_50_ = 0.636 μM; Cpd-60: EC_50_ = 1.50 μM).

**Table 4:** Antiproliferative Activity Assay (MTT) in MV-3 and MDA-MB-231 Cells^a^

	MV-3 EC_50_ [μM]^b^			MDA-MB-231 EC_50_ [μM]^b^		
incubationtime	72 h	96 h	120 h	72 h	96 h	120 h
**ST13**	n.d.^c^	7.94 ± 1.36	2.77 ± 0.88	n.d.^c^	13.9 ± 3.4	4.87 ± 0.86
Cpd-60	n.d.^c^	2.34 ± 0.79	0.37 ± 0.04	n.d.^c^	3.21 ± 1.01	0.89 ± 0.25
vorinostat	1.88 ± 0.73	1.36 ± 0.36	1.23 ± 0.35	1.73 ± 0.19	1.23 ± 0.22	1.11 ± 0.17

a Cells were incubated for 72, 96, and 120 h with **ST13**, Cpd-60, and vorinostat.

b IC_50_ values are reported as mean ± SD from at least three independent experiments.

c n.d.: EC_50_ not determined, viability reduction at 15 μM: <25%.

### Effect on Apoptosis Induction

To further investigate the mechanism of cell death induced by **ST13** and Cpd-60, an annexin V/propidium iodide (PI) apoptosis assay was performed. As shown in Figure 7, **ST13** and Cpd-60 significantly induced apoptosis in MDA-MB-231 cells.

**Figure 7:**
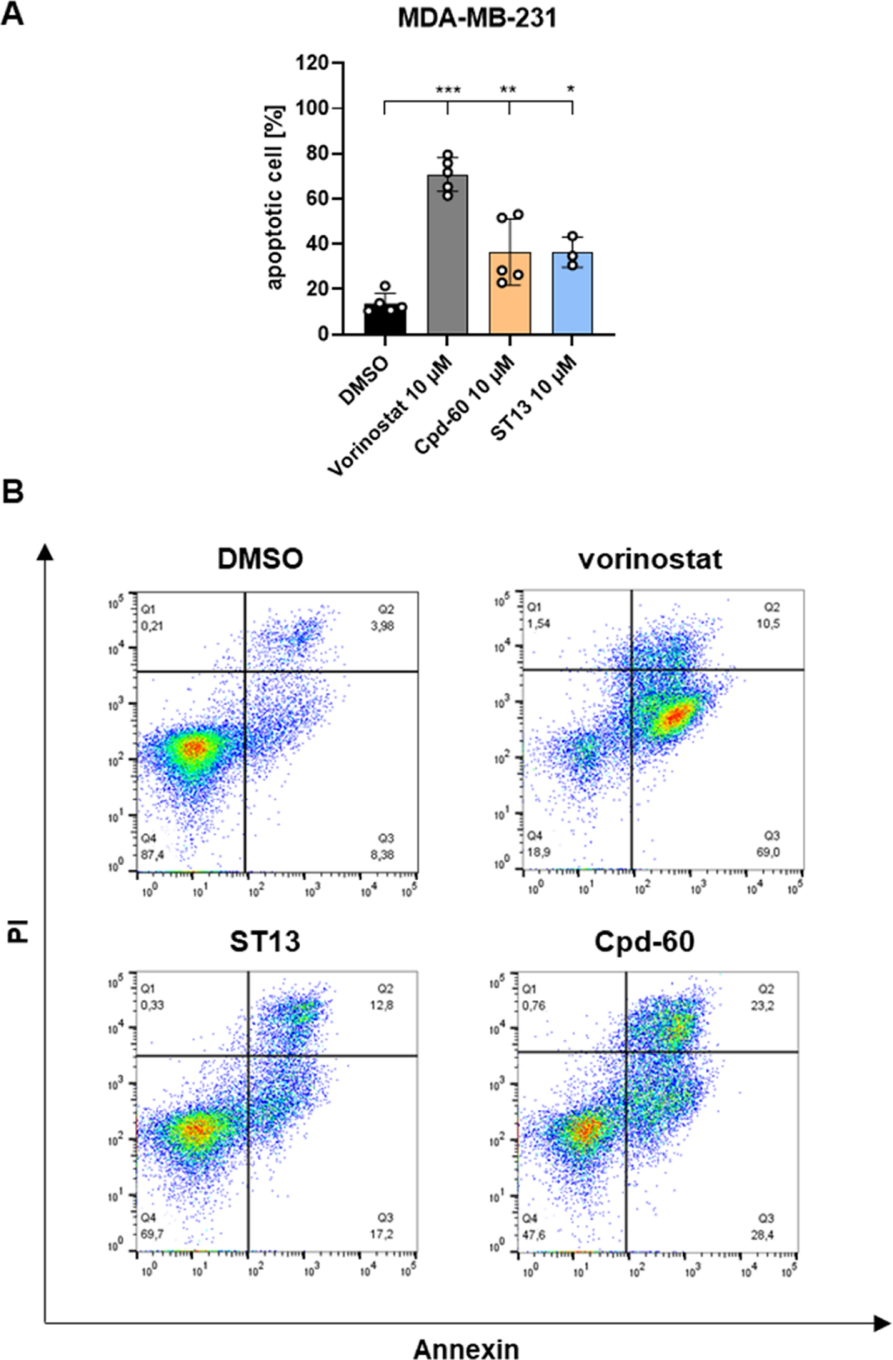
Results from annexin V/PI apoptosis assay. MDA-MB-231 cells were treated with vehicle (DMSO) or 10 μM of vorinostat, Cpd-60, or **ST13** for 48 h. Cells were stained with annexin V and propidium iodide (PI). (A) Data represents the mean ± SD of at least three individual experiments. Significances were determined by one-way ANOVA followed by Dunnett’s multiple comparisons test and are indicated by asterisks (*P ≤ 0.05, **P ≤ 0.01, ***P ≤ 0.001). (B) Representative Annexin V/PI dot plots showing apoptosis induction.

### Design and Synthesis of a Photocaged 2-Hydroxyanilide-Based HDAC Inhibitor

Phenolic groups are well-suited for photocaging strategies.^27,42^ We therefore hypothesized that **ST13** could be efficiently converted into a photocaged prodrug by masking the phenol group of its *ortho*-hydroxyanilide ZBG, thereby potentially enabling spatiotemporal control of HDAC1/HDAC2 inhibition. To this end, **ST13** was converted into the light-activatable prodrug **ST17** (see Scheme 1) by introducing the well-established 4,5-dimethoxy-2-nitrobenzyl (DMNB) moiety as a photoremovable protecting group.^43,44^ The DMNB group is particularly suitable for our intended application due to its excellent quantitative cleavage and low inherent toxicity.^33,43,44^ The photocaged derivative **ST17** was obtained by alkylation of **ST13** via a Williamson ether synthesis using 1-(bromomethyl)-4,5-dimethoxy-2-nitrobenzene as alkylating agent and K_2_CO_3_ (Scheme 1).

### Characterization of the Photocaged HDAC Inhibitor **ST17**

The deprotection of **ST17** and its effective conversion (82%) to its parent HDAC inhibitor **ST13** upon irradiation (10 min; λ = 365 nm) was confirmed by high-performance liquid chromatography (HPLC) experiments (see Figure S3, Supporting Information). Next, **ST17** was tested for its inhibitory activity against HDAC1, HDAC2, HDAC3, and HDAC6 with and without irradiation at 365 nm (see Table 5). As expected, light-treated **ST17** (HDAC1: IC_50_ = 0.040 μM; HDAC2: IC_50_ = 0.072 μM) showed potent HDAC1/HDAC2 inhibition comparable to the parent compound **ST13** (HDAC1 IC_50_ = 0.023 μM; HDAC2: IC_50_ = 0.049 μM). Surprisingly, the nonirradiated prodrug **ST17** also demonstrated some inhibitory activity at HDAC1 and HDAC2. However, although **ST17** does inhibit HDAC1 and HDAC2, its potency is considerably lower than that of **ST13** (HDAC1: IC_50_ = 0.840 μM vs 0.023 μM; HDAC2: IC_50_ = 1.71 μM vs 0.049 μM). Notably, this phenomenon has also been observed for other HDAC prodrugs, such as carbamates.^45^

**Table 5:** Inhibitory Activity of Photocaged **ST17** (with and without UV Irradiation), **ST13**, and Vorinostat against HDAC1, HDAC2, HDAC3, and HDAC6

	IC_50_ [μM]			
	HDAC1^a^ ^,^ ^b^	HDAC2^a^ ^,^ ^b^	HDAC3^a^ ^,^ ^b^	HDAC6^a^ ^,^ ^c^
**ST17**	0.840 ± 0.308	1.71 ± 0.83	>10^d^	>10^d^
**ST17** + UV	0.040 ± 0.008	0.072 ± 0.012	8.58 ± 0.76	>10^d^
**ST13**	0.023 ± 0.003	0.049 ± 0.003	6.64 ± 2.18	>10^d^
vorinostat	0.111 ± 0.009	0.207 ± 0.021	0.107 ± 0.022	0.056 ± 0.008

a Mean ± standard deviation (SD), at least two independent experiments.

b Preincubation of enzyme and inhibitor: 1 h at RT.

c Preincubation of enzyme and inhibitor: 15 min at RT.

d <30% inhibition at the stated concentration.

Furthermore, **ST17** was assessed in a cellular environment using the whole cell HDAC assay and cell viability assays. The results are summarized in Table 6. In the cellular HDAC inhibition assay, light-treated **ST17** showed nanomolar IC_50_ values against both MV-3 and MDA-MB231 cells (MV-3: IC_50_ = 0.392 μM; MDA-MB231: IC_50_ = 0.783 μM) similar to **ST13** (MV-3: IC_50_ = 0.311 μM; MDA-MB231: IC_50_ = 0.577 μM). Notably, light-induced activation of **ST17** resulted in a 15-fold increase of activity in MV-3 cells and a 4.5-fold increase in MDA-MB-231 cells compared to untreated **ST17**, further confirming **ST17** as a photocleavable HDAC inhibitor prodrug. Furthermore, **ST17** was evaluated using a MTT viability assay against MV-3 and MDA-MB-231 cells; the results are summarized in Table 6 (right). Similarly, we observed considerably lower antiproliferative activity for untreated **ST17** (MV-3: EC_50_ = 16.2 μM; MDA-MB-231: EC_50_ = 11.2 μM), while light-treated **ST17** (MV-3: EC_50_ = 4.64 μM; MDA-MB-231: EC_50_ = 6.78 μM) demonstrated low micromolar activity comparable to the parent compound **ST13** (MV-3: EC_50_ = 2.77 μM; MDA-MB-231: EC_50_ = 4.87 μM).

**Table 6:** Inhibitory Activity (Whole-Cell HDAC Inhibition Assay, Left) and Antiproliferative Activity (MTT Cell Viability Assay, Right) of Photocaged **ST17** (with and without UV Irradiation), **ST13**, and Vorinostat against the MV-3 and MDA-MB-231 Cancer Cell Lines

	whole-cell HDAC inhibition assay EC_50_ [μM]^a^		MTT cell viability assay EC_50_ [μM]^a^ ^,^ ^b^	
	MV-3	MDA-MB-231	MV-3	MDA-MB-231
**ST17**	5.98 ± 1.24	3.55 ± 2.00	16.2 ± 4.1	11.2 ± 1.7
**ST17** + UV	0.392 ± 0.070	0.783 ± 0.139	4.64 ± 0.93	6.78 ± 0.58
**ST13**	0.311 ± 0.047	0.577 ± 0.097	2.77 ± 0.88	4.87 ± 0.86
vorinostat	0.406 ± 0.069	0.240 ± 0.053	1.23 ± 0.35	1.11 ± 0.17

a EC_50_ values are reported as mean ± SD from at least three independent experiments.

b 120 h preincubation of cells and inhibitor.

## Conclusion

Pan-HDAC inhibitors like vorinostat frequently cause severe adverse side effects, because they inhibit multiple HDAC isoforms. This issue has directed research toward more selective HDACi, which is often facilitated by choosing nonhydroxamate ZBGs. Selective HDAC1/HDAC2 inhibitors are particularly interesting in this context, as they exhibit profound anticancer activity, but may carry a reduced risk of dose-limiting adverse effects. In this study, we characterized two structurally distinct class I selective HDAC1/HDAC2 inhibitors: **ST13**, based on an *ortho*-hydroxyanilide scaffold, and Cpd-60, featuring the well-established *ortho*-aminoanilide ZBG. Both compounds exhibited potent and selective inhibition of HDAC1 and HDAC2 *in vitro*, with IC_50_ values in the low nanomolar range and minimal activity against other isoforms. In biochemical assays, we investigated the binding kinetics of **ST13** and Cpd-60, identifying **ST13** as a slow- and tight-binding HDAC1/HDAC2 inhibitor that binds to HDAC1 *via* an “induced fit” mechanism similar to Cpd-60. These results were further validated in cellular assays: The delay in histone H3 hyperacetylation and the sustained effects after compound wash out are further evidence of the slow-acting and durable HDAC inhibitory properties of both **ST13** and Cpd-60. As a result, **ST13** represents a promising alternative to *ortho*-aminoanilide-based inhibitors such as Cpd-60, offering comparable efficacy and binding behavior. We also demonstrated the functional effects of these inhibitors in cancer cell models, showing antiproliferative activity and apoptosis induction, with Cpd-60 being more potent overall. Interestingly, both compounds exhibited a remarkable time-dependent antiproliferative effects, emphasizing the importance of considering slow-binding kinetics when assessing inhibitor potency in cellular assays. Overall, selective slow- and tight-binding HDAC inhibitors are very promising. Prolonged target engagement is advantageous, because it leads to more durable pharmacological effects *in vivo*. This allows for longer dosing intervals, and can also reduce off-target toxicity.^38,46^ Notably, since toxicity has limited the therapeutic application of first-generation HDAC inhibitors in oncology^8^ and beyond, selective, slow- and tight-binding HDAC inhibitors may provide a safer and more tolerable alternative.

Additionally, we extended our investigation to a photopharmacological approach by designing **ST17**, a photocaged prodrug of **ST13**. This initial proof-of-concept study aimed to demonstrate the feasibility of light-triggered activation of a selective HDAC inhibitor in vitro. **ST17** showed low activity in its protected form but was efficiently converted to the active inhibitor upon light exposure, enabling selective and controllable HDAC1/HDAC2 inhibition in both biochemical and cellular assays. With **ST17**, we demonstrate that a selective HDAC inhibitor bearing an *ortho*-hydroxyanilide ZBG can be easily converted into a photocaged compound. The DMNB group can be attached to the phenolic hydroxyl group in one step. In contrast, previous attempts to attach the DMNB group via a carbamate linkage to an *ortho*-aminoanilide-based inhibitor did not allow efficient photoactivation under comparable conditions.^26^ Collectively, our results establish a robust framework for the development of *ortho*-hydroxyanilide-based HDAC inhibitors and highlight their interesting potential for photopharmacological applications. Looking ahead, future efforts could focus on the optimization of the photocager design by using photoremovable protecting groups that are activated by light of longer wavelength, which is more suitable for deep tissue penetration and *in vivo* studies, or by introducing photoswitches that enable reversible regulation.^28^ Another interesting approach could be to employ photoresponsive drug delivery systems that allow controlled release of an active substance from a light-sensitive carrier system.^28^

## Methods

### HPLC Photolysis Experiments

To initiate photolysis, the photocaged HDAC inhibitor **ST17** was irradiated at room temperature using a UVP-CL-1000 Cross-linker (Analytik Jena GmbH, Jena, Germany) equipped with five-UV-A tubes (F8T5; λ_max_ = 365 nm; Analytik Jena GmbH, Jena, Germany). For the deprotection experiments, 10 μL of a 1 mM **ST17** solution in DMSO were irradiated for 10 min. Subsequently, samples were diluted with acetonitrile to a final concentration of 100 μM and analyzed via HPLC/UV (λ_quant_ = 250 nm) using the area under the curve (area [mAU × min]) for quantification. The amount of **ST13** released from the photocager **ST17** was quantified using a calibration curve of **ST13** (see Figure S2).

### HDAC Enzyme Inhibition Assay

First, 3-fold serial dilutions of test compounds were prepared in DMSO. Predilutions designated for photolysis were irradiated for 10 min using a UVP CL-1000 Cross-linker (Analytik Jena GmbH, Jena, Germany) described above. Next, 1.0 μL of these serial dilutions were added to 29 μL assay buffer (50 mM Tris-HCl, pH 8.0, 137 mM NaCl, 2.7 mM KCl, 1 mM MgCl2, 0.1 mg/mL BSA) into black 96-well microplates (OptiPlate-96 Black, PerkinElmer, Waltham, MA, USA). Then, 10 μL of human recombinant HDAC1 (BPS Bioscience Inc., San Diego, CA, USA, #50051), HDAC2 (BPS Bioscience Inc., San Diego, CA, USA, #50052), HDAC3 (BPS Bioscience Inc., San Diego, CA, USA, #50053), or HDAC6 (PBS Bioscience Inc., San Diego, CA, USA, #50006) enzyme solution in assay buffer were added, followed by an incubation period of 60 min at room temperature. Next, 10 μL of the fluorogenic substrate ZMAL^26^ (Z-Lysin­(Ac)-AMC) was added and incubated for further 90 min at 37 °C. Finally, 50 μL 0.4 mg/mL trypsin in trypsin buffer (50 mM Tris-HCl, pH 8.0, 100 mM NaCl) was added an incubated for additional 30 min at 37 °C. The final assay volume of 100 μL contained following enzyme concentrations: 130 pg/μL HDAC1, 95 pg/μL HDAC2, 100 pg/μL HDAC3, and 330 pg/μL HDAC6. Fluorescence (excitation: 355 nm, emission: 460 nm) was measured using a FLUOstar OPTIMA microplate reader (BMG LABTECH GmbH, Ortenberg, Germany). IC_50_ values were determined by generating normalized dose–response curves using the three-parameter logistic equation (GraphPad Prism 8.0, San Diego, USA). All compounds were tested in duplicates and IC_50_ values were calculated from at least two independent experiments.

### IC_50_-Shift Experiments at HDAC1 and HDAC2

For IC_50_-shift experiments the preincubation time for the HDAC enzyme inhibition assay was varied. HDAC1 and HDAC2 and the test compounds were preincubated for 5, 30, 60, or 120 min at room temperature. Afterward, the assay was continued as described in the HDAC enzyme inhibition assay. Compounds were tested in duplicates, IC_50_ values were calculated from at least three independent experiments.

### 100-Fold Jump-Dilution Experiments at HDAC1 and HDAC2

Initially, HDAC1 (26 ng/μL) and test compounds (5× or 10× IC_50_) or vehicle were incubated in an “incubation mix” in assay buffer in PCR tubes for 60 min at room temperature. Afterward, the “incubation mix” was diluted 100-fold either with assay buffer or the respective inhibitor solution at its original concentration. Then, 20 μL ZMAL solution (250 μM) in assay buffer and 10 μL trypsin solution (1000 ng/μL) in trypsin buffer were added to a final volume of 100 μL. Fluorescence (excitation: 355 nm, emission: 460 nm) was measured continuously for 40 min at 28 °C at a Spark microplate reader (Tecan Group AG, Maennedorf, Swiss). Compounds were tested in triplicates in at least two independent experiments.

For HDAC2, test compounds were incubated with HDAC2 (19 ng/μL) at 28 °C. ZMAL was used in a concentration of 62.5 μM and trypsin in a concentration of 1300 ng/μL.

### Determination of Binding Kinetics via the *Progression Method*

Appropriate test compound concentrations were chosen based on the previously determined IC_50_ values. Ten μL HDAC1 solution in assay buffer (2.6 ng/μL) were mixed with 20 μL ZMAL solution (250 μM in assay buffer), 10 μL test compound in assay buffer, 10 μL trypsin solution (1000 ng/μL in trypsin buffer) and 50 μL assay buffer and fluorescence was monitored continuously for 45 min at 28 °C. Each progression curve was fitted to either obtain the apparent first-order rate constant *k*_obs_ (eq 1) or the linear conversion rates *v*_i_ and *v*_0_ (eq 2). For fast-on/fast-off inhibitors, the ratio *v*_i_/*v*_0_ was plotted against the corresponding inhibitor concentrations. For vorinostat the curve was fitted to eq 3 to determine the *K*_i_ value. For slow-binding inhibitors *k*_obs_ was plotted against the corresponding inhibitor concentration and fitted to eq 4. Compounds were tested in triplicates in at least two independent experiments.

1 $$\left[P\right]=v_{ss}t+\frac{v_{in}-v_{ss}}{k_{obs}}\left(1-e^{-k_{obs}t}\right)$$

Equation 1 describes the relationship between the concentration of AMC [*P*] and the time and is employed to determine the *k*_obs_ value of each concentration of a slow binding inhibitor.

2 $$\left[P\right]=v_{ss}t$$

Equation 2 describes the relationship between the concentration of AMC [*P*] and the time for a fast-on/fast-off binding inhibitor.

3 $$\frac{v_{i}}{v_{0}}=\frac{1}{\frac{\left[I\right]}{K_{i}\left(1+\frac{\left[S\right]}{K_{m}}\right)}+1}$$

Equation 3 describes the relationship between *v*_i_/*v*_0_ and the concentration of a fast-on/fast-off inhibitor and allows the determination of *K*_i_.

4 $$k_{obs}=k_{-2}+\frac{k_{2}}{\left[I\right]+K_{i,1}\left(1+\frac{\left[S\right]}{K_{M}}\right)}\left[I\right]$$

Equation 4 describes the relationship between *k*_obs_ and the concentration of a slow binding inhibitor [*I*] with a mechanism *I* (“induced fit”) binding mode. *k*_–2_ describes *k*_off_, *k*_2_ describes *k*_on_ and *K*_i,1_ is the initial inhibitor–enzyme complex [EI].

### *K*_m_ Determination for HDAC1 and HDAC2

The *K*_m_ was determined using a series of ZMAL concentrations as shown in Figure S4. To this end, 20 μL of ZMAL (in assay buffer), 10 μL of trypsin solution (1000 ng/μL (HDAC1) or 1300 ng/μL (HDAC2) in trypsin buffer), 10 μL enzyme solution (2.6 ng/μL (HDAC1) or 1.9 ng/μL (HDAC2) in assay buffer) and 60 μL assay buffer were pipetted into black 96-well microplates (OptiPlate-96 Black, PerkinElmer, Waltham, MA, USA). Fluorescence (excitation: 355 nm, emission: 460 nm) was measured continuously using a Spark microplate reader (Tecan Group AG, Maennedorf, Swiss). The respective steady-state velocities were plotted against the corresponding substrate concentrations [*S*] and fitted to the *Michaelis Menten* equation. To ensure substrate excess during continuous assays (see above), the substrate concentration was set to five times *K*_m_.

### Cell Culture

The human melanoma cell line MV-3 was obtained from EPO GmbH Berlin-Buch and was cultivated in RPMI-1640 medium (PAN Biotech GmbH; Aidenbach, Germany) supplemented with 100 U/mL penicillin, 100 U/mL streptomycin (*PAN Biotech* GmbH; Aidenbach, Germany) and 10% FBS (*PAN Biotech* GmbH; Aidenbach, Germany). The human breast cancer cell line MDA-MB-231 was obtained from ATCC (Manassas, VA, USA) and was cultivated in DMEM medium (*PAN Biotech* GmbH; Aidenbach, Germany) supplemented with 100 U/mL penicillin, 100 U/mL streptomycin (PAN Biotech GmbH; Aidenbach, Germany), 2 mM l-glutamine (*PAN Biotech* GmbH; Aidenbach, Germany) and 1 mM sodium pyruvate (*ThermoFisher Scientific* Inc.; Waltham, MA, USA). Both cell lines were cultivated at a humidified atmosphere at 37 °C containing 5% CO_2_. Mycoplasma contamination was routinely excluded by PCR.

### Cell Viability Assay

MTT (3-(4,5-dimethylthiazol-2-yl)-2,5-diphenyltetrazolium bromide; Catalog# A2231; *BioChemica*, Applichem GmbH, Darmstadt, Germany) was utilized to measure the antiproliferative effect. Depending on the incubation time, 3000 MV-3 cells (72 h), 2000 MV-3 cells (96 h) or 1000 MV-3 cells (120 h) per well were seeded in 96-well plates (*Starlab GmbH*, Hamburg, Germany) with each well containing 199 μL of volume. For MDA-MB-231 the seeding densities were 8000 cells per well for a 72 h incubation period, 4000 cells per well for 92 h and 3000 cells per well for 120 h. Next, predilutions of the test compounds were prepared in DMSO. Predilutions designated for photolysis were irradiated for 10 min with a UVP CL-1000 Cross-linker (Analytik Jena GmbH, Jena, Germany; see above). Subsequently, cells were treated with 1.0 μL of the dilution series of the test compounds. Following an incubation period of 72, 96, or 120 h, 40 μL of freshly prepared MTT solution (5 mg/mL in PBS) was added and incubated for 1 h at 37 °C and 5% CO_2_. After removing the supernatant, formazan was solubilized in 200.0 μL DMSO. The absorbance was measured at 570 nm with background subtraction at 690 nm using a Multiskan microplate photometer (*Thermo Fisher Scientific*, Waltham, MA, USA). The obtained data were normalized to PBS, considering 100% viability. EC_50_ values were determined by generating normalized dose–response curves using the four-parameter logistic equation (*GraphPad* Prism 8.0, San Diego, USA). All compounds were tested in triplicates and EC_50_ values were calculated from at least three independent experiments.

### The CellTiter-Glo 2.0 Cell Viability Assay

(*Promega*; Walldorf, Germany) was utilized to assess the antiproliferative effects. MM.1S and HEK293 cells were seeded at a density of 1000 cells per well in 384-well plates, with each well containing 22.5 μL of volume. The test compounds were first dissolved in DMSO and then diluted at a ratio of 1:20 using cell culture medium. Subsequently, cells were stimulated with 2.5 μL of the dilution series of the test compounds. Following compound exposure, the plates were incubated for 120 h in the incubator set at 37 °C with 5% CO_2_. Post 120 h of incubation, 25.0 μL of CellTiter-Glo reagent was dispensed into each well and the plates were incubated at the room temperature for 10 min. Luminescence was then measured using a Spark multimode microplate reader (Tecan Group AG, Maennedorf, Swiss). Data were normalized against controls: wells treated with 0.5% DMSO were set as 100% viable, while those treated with 10% DMSO were considered completely nonviable (0% viability). EC_50_ values were calculated by fitting normalized dose–response curves using the four-parameter logistic equation (*GraphPad* Prism 8.0, San Diego, USA). All compounds were tested in duplicates and EC_50_ values were calculated from at least two independent experiments. Compounds exhibiting a viability of less than 50% at a concentration of 50 μM were considered nontoxic.

### Immunoblot Analysis

Depending on the incubation time, MV-3 cells were seeded to T25 cell culture flasks at a density of 0.2 × 10^6^ cells/mL (24 h), 0.1 × 10^6^ cells/mL (48 h) or 0.05 × 10^6^ cells/mL (72 h) and were allowed to adhere for 24 h. After that, cells were treated with a concentration of 5 μM of HDAC inhibitor or vehicle (DMSO) for additional 24 h, 48 h or 72 h. For cell lysis the cells were initially washed with PBS and detached using a scraper. The cells were then centrifuged at 1200 rpm for 4 min at 4 °C, followed by two additional washing steps. After that, the cell pellet was resuspended in 100 μL of freshly prepared lysis buffer and incubated on ice for 30 min with vortexing every 10 min. The lysis buffer consisted of Invitrogen cell extraction buffer (10 mM Tris, pH 7.4, 100 mM NaCl, 1 mM EDTA, 1 mM EDTA, 1 mM NaF, 20 mM Na_4_P_2_O_7_, 2 mM Na_3_VO_4_, 1% Triton × −100, 10% glycerol, 0.1% SDS, 0.5% deoxycholate; Catalog# FNN0011, *Thermo Fisher Scientific Inc.*, Waltham, MA, USA), supplemented with 0.1 mM PMSF (Catalog# 10837091001, *Sigma-Aldrich*, St. Louis, MO, USA) and Halt Protease Inhibitor (100×) (Catalog# 78429, *Life Technologies GmbH* Carlsbad, CA, USA). Finally, the samples were centrifuged at 14,000 rpm and 4 °C for 30 min and the supernatant was collected. Protein quantification was performed by using BCA protein assay reagents (Catalog# 23225, *Thermo Fisher Scientific Inc.*, Waltham, MA, USA). Lysates were mixed with Laemmli sample buffer 2× concentrate (Catalog# S3401-10VL, *Sigma-Aldrich*, St. Louis, MO, USA) to a protein concentration of 1.25 μg/μL and denatured at 95 °C for 5 min. Subsequently, SDS-PAGE was performed with 2–20% Mini-PROTEAN TGX stain-free gels (Catalog# 456809, *Bio-Rad*, Hercules, CA, USA) at 200 V for 45 min. Proteins were then transferred to a Trans-Blot Turbo-PVDF membrane (Catalog# 1704156, *Bio-Rad*, Hercules, CA, USA) with the Trans-Blot Turbo Transfer System (Catalog# 1704150, *Bio-Rad*, Hercules, CA, USA) at 25 V for 5 min. The membrane was blocked with a solution of 5% milk powder in TBST (Tris-buffered saline-Tween 20 0.2%) for 60 min at room temperature. After three washing cycles for 10 min with TBST, the membrane was incubated with the respective antibody and a mouse anti-GAPDH antibody (Catalog# T0004, *Affinity Biosciences*, Cincinnati, OH, USA; 1:20,000 dilution) at room temperature for 60 min and 4 °C overnight. For detection of acetylation levels of histone H3 and α-tubulin, antibodies specific for acetyl-histone H3 (Catalog# 06-599, *Millipore*, Darmstadt, Germany; 1:1000 dilution) and antiacetyl-α-tubulin (Lys40) (Catalog# 5335, *Cell Signaling Technology*, Denver, MA, USA: 1:1000 dilution) were used as primary antibodies. Membrane was rinsed again three times before applying the secondary antibody for 1.5 h at room temperature. As secondary antibody HRP-conjugated antimouse IgGκ binding protein (Catalog# sc-516102, *Santa Cruz*, Dallas, TX, USA; 1:10,000 dilution) and HRP-conjugated antirabbit IgG antibody (Catalog# HAF008, *R&D Systems, Inc.*, Minneapolis, MN, USA; 1:10,000 dilution) were employed. After washing off the secondary antibody, protein bands were detected via chemiluminescence using Clarity Western ECL substrate (Catalog# 1705061, *Bio-Rad*, Hercules, CA, USA). Detection and Analysis were performed with ChemiDox XRS + System (Catalog# 1708265, *Bio-Rad*, Hercules, CA, USA) and Image Lab software v. 6.1 (*Bio-Rad*, Hercules, CA, USA).

For wash out experiments, MV-3 cells were gently washed twice with 1 mL PBS after a preincubation period of 48 h. Subsequently MV-3 cells were cultured in FBS-free medium for either 0, 3, or 6 h before lysis.

### HDAC Cell Inhibition Assay

MV-3 cells and MDA-MB-231 cells were seeded in a concentration of 15 × 10^3^ cells/well (total volume of 89.5 μL) in 96-well cell culture microplates (Catalog# 655086, *Greiner Bio-One*) and were allowed to adhere for 24 h. Afterward, cells were treated with dilution series of the test compounds. For this purpose, 3-fold serial dilutions of test compounds (DMSO) were prepared and added at a volume of 0.5 μL to the cells. Predilutions designated for photolysis were irradiated for 10 min using a UVP CL-1000 Cross-linker (Analytik Jena GmbH, Jena, Germany; see above). After an incubation period of 18 h the fluorogenic substrate MAL (Boc-Lys­(ε-Ac)-AMC; Catalog# 233691-67-3, *BLD pharma*) was added and cells were incubated for 3 h at 37 °C and 5% CO_2_. MAL was used in a final concentration of 0.3 mM in the respective cell culture medium with 0.5% IGEPAL CA-630 (Catalog# J61055, *Alfa Aesar*). Subsequently, 100 μL of stop solution (50 mM Tris-HCl, 137 mM NaCl, 2.7 mM KCl, 1 mM MgCl_2_, 1% IGEPAL CA-630, 10 μM vorinostat, 2.0 mg/mL trypsin) was added, following 1.5 h incubation at 37 °C and 5% CO_2_. Fluorescence (excitation λ = 355 nm, emission λ = 460 nm) was measured using a Spark microplate reader (Tecan Group AG, Maennedorf, Swiss). The obtained data were normalized to vehicle treated cells, considering 100% enzyme activity. IC_50_ values were determined by generating dose–response curves using the four-parameter logistic equation (*GraphPad* Prism 8.0, San Diego, USA). All compounds were tested in duplicates and EC_50_ values were calculated from at least three independent experiments.

### Annexin V/PI Assay

MDA-MB-231 cells were seeded in 6-well plates at a density of 0.125 × 10^6^ cells/well (72 h) and treated with the respective test compound or vehicle (DMSO). After the incubation period, cells were collected and washed with cell staining buffer (HEPES 0.1 M, NaCl 1.4 M, CaCl_2_ × 3 H_2_O 25 mM). MDA-MB-231 cells were stained with 5 μL/well annexin V-Pacific blue (catalog# 640945, BioLegend, San Diego, CA, USA) and 10 μL/well propidium iodide (catalog# 421301, BioLegend, San Diego, CA, USA), incubated for 15 min and measured at the Guave EasyCyte 11HT (Guava easyCyteTM, Luminex, Austin, TX, USA). Data was evaluated with GraphPad Prism 8 (GraphPad Software, San Diego, CA, USA).

### Molecular Docking

The structures of the ligands were drawn with the software ChemDraw 21.0.0.28 (PerkinElmer Inc., Waltham, MA, USA) and prepared in the software Molecular Operating Environment (MOE, Chemical Computing Group, version 2024.06)^47^ by generating the 3D structure and energy minimization using AMBER:EHT force field. The prepared ligands were used as input for AutoDock tools version 1.5.6. The crystal structure of HDAC2 (PDB: 3MAX)^48^ was downloaded from the Protein Data Base. The native ligand, buffer, noninteracting ions, and all waters molecules were removed. AutodockTools-1.5.6 program^49^ was used to add all hydrogen atoms, modify histidine protonation (H145 and H146, adding only HD1),^50^ compute gasteiger charges, and merge all nonpolar hydrogens. After the generation of the pdbqt output file, the charge of the zinc atom was manually changed to +2. Autogrid 4.2.6 ^49^ was utilized to generate the grid files using a grid box with the following settings: spacing of 0.375 Å, grid box size of 40 × 40 × 40 centered around the coordinates of the native ligand. Autodock 4.2.6 ^49^ was used to perform the docking calculations. The generated pdbqt file of the enzyme was set as a rigid macromolecule. The genetic algorithm search parameters were set to 100 GA runs for each ligand with a population size of 150, a maximum number of 2.5 × 10^6^ energy evaluations, a maximum number of 2.7 × 10^4^ generations, a mutation rate of 0.02, and a crossover rate of 0.8. The lowest-energy conformer of the first cluster showing Zn^2+^ binding was used as the representative predicted binding pose.

## Chemistry

### General Remarks

Chemicals were obtained from BLDpharm, Sigma-Aldrich, TCI Chemicals, or abcr GmbH and used without purification. Air-sensitive reactions were carried out under argon atmosphere utilizing standard *Schlenk* techniques. Thin-layer chromatography (TLC) was carried out on prefabricated plates (silica gel 60, F_254_, Merck). Components were visualized by irradiation with ultraviolet light (254 nm). Column chromatography was carried out on silica gel (60 Å, 40–60 μm, *Acros Organics*).

Nuclear magnetic resonance spectroscopy (NMR): Proton (^1^H) and carbon (^13^C) NMR spectra were recorded either on a Bruker AvanceDRX 500 (500 MHz ^1^H NMR, 126 MHz ^13^C NMR) or a BrukerAvance III 600 (600 MHz ^1^H NMR, 151 MHz ^13^C NMR). The chemical shifts are given in parts per million (ppm). Deuterated dimethyl sulfoxide (DMSO-*d*_6_) was used as solvent.

High performance liquid chromatography (HPLC): A *Thermo Fisher Scientific* UltiMate 3000 UHPLC system with a Nucleodur 100-5 C18 (250 mm × 4.6 mm, *Macherey Nagel*) with a flow rate of 1 mL/min and a temperature of 25 °C or a 100-5 C18 (100 mm × 3 mm, *Macherey Nagel*) with a flow rate of 0.5 mL/min and a temperature of 25 °C with an appropriate gradient were used. The purity of all final compounds was 95% or higher. Purity was determined via HPLC with the Nucleodur 100-5 C18 (250 mm × 4.6 mm, *Macherey Nagel*) at 250 nm.

Flash chromatography was performed on an Interchim puriFlash XS 520 Plus with a diode-array detector (DAD) from 200 to 400 nm using prepacked silica gel cartridges (PF-30SIHP-F0012-F0040) or C18 reversed-phase cartridges (PF-30C18HP-F0004-F0012).

### Synthesis of **ST01** and **ST13**

To a mixture of 4-acetamidobenzoic acid (179 mg, 1.0 mmol, 1.0 equiv) and DIPEA (258 mg, 2.0 mmol, 2.0 equiv) in anhydrous DMF (20 mL) was added HATU (570 mg, 1.5 mmol, 1.5 equiv) and the reaction mixture was stirred at room temperature for 30 min. Then, 2-aminophenol (109 mg, 1.0 mmol, 1.0 equiv) or 3-amino-[1,1′-biphenyl]-4-ol (185 mg, 1.0 mmol, 1.0 equiv) was added, and the mixture was stirred at room temperature for 16 h. Afterward, the solvent DMF was removed under vacuum. The obtained residue was purified by flash column chromatography (C18 reversed phase, MeCN/H_2_O 5–95%) to obtain **ST01** and **ST13**.

#### 4-Acetamido-*N*-(2-hydroxyphenyl)­benzamide (**ST01**)

White solid, 54 mg, yield 20%. ^1^H NMR (ppm, 600 MHz, DMSO-*d*_6_): δ = 10.21 (s, 1H), 9.72 (s, 1H), 9.41 (s, 1H), 7.93–7.92 (m, 2H), 7.72–7.71 (m, 2H), 7.69 (dd, *J* = 1.0, 1.0 Hz, 1H), 7.04–7.01 (m, 1H), 6.91 (dd, *J* = 1.0, 1.0 Hz, 1H), 6.85–6.81 (m, 1H), 2.09 (s, 3H); ^13^C NMR (ppm, 126 MHz, DMSO-*d*_6_): δ = 169.2, 165.2, 149.6, 142.9, 128.9, 128.9, 126.6, 125.9, 124.3, 119.6, 118.7, 116.6, 24.6. LRMS-ESI (*m*/*z*): [M + H]^+^ calcd for C_15_H_15_N_2_O_3_, 271.1; found, 271.2; HPLC: *t*_R_ = 11.67 min (96.5% purity).

#### 4-Acetamido-*N*-(4-hydroxy-[1,1′-biphenyl]-3-yl)­benzamide (**ST13**)

Light yellow solid, 45 mg, yield 13%. ^1^H NMR (ppm, 500 MHz, DMSO-*d*_6_): δ = 10.21 (s, 1H), 9.95 (s, 1H), 9.49 (s, 1H), 8.04 (d, *J* = 2.5 Hz, 1H), 7.97–7.95 (m, 2H), 7.74–7.72 (m, 2H), 7.58 (d, *J* = 8.0 Hz, 2H), 7.43 (q, *J* = 6.5 Hz, 2H), 7.35 (dd, *J* = 2.5, 2.5 Hz, 1H), 7.32–7.29 (m, 1H), 7.01 (d, *J* = 8.5 Hz, 1H) and 2.10 (s, 3H); ^13^C NMR (ppm, 151 MHz, DMSO-*d*_6_): δ = 169.0, 165.0, 149.0, 142.6, 140.2, 131.4, 129.0, 128.7, 128.5, 126.8, 126.6, 126.2, 123.9, 122.2, 118.4, 116.7, 24.3; HRMS-ESI (*m*/*z*): [M + H]^+^ calcd for C_21_H_18_N_2_O_3_, 347.1390; found, 347.1392; HPLC: *t*_R_ = 12.58 min (96.9% purity).

#### 4-Acetamido-*N*-(4-((4,5-dimethoxy-2-nitrobenzyl)­oxy)-[1,1′-biphenyl]-3-yl)­benzamide (**ST17**)

**ST13** (346 mg, 1.0 mmol, 1.0 equiv), 1-(bromomethyl)-4,5-dimethoxy-2-nitrobenzene (552 mg, 2.0 mmol, 2.0 equiv), and K_2_CO_3_ (553 mg, 4.0 mmol, 4.0 equiv) were dissolved in acetone (20 mL). The obtained solution was stirred at 60 °C for 12 h. Afterward, the reaction mixture was cooled and the solvent was removed by evaporation. The residue was treated with H_2_O (15 mL) and extracted with ethyl acetate (3 × 15 mL). The organic phase was washed with water, dried over Na_2_SO_4_, and evaporated under vacuum. The crude product was purified by silica gel chromatography (DCM/methanol = 40:1, v/v) to obtain the final product **ST17** as a light-yellow solid, 233 mg, yield 43%. ^1^H NMR (ppm, 600 MHz, DMSO-*d*_6_): δ = 10.21 (s, 1H), 9.72 (s, 1H), 7.95 (s, 2H), 7.94 (s, 1H), 7.73 (s, 1H), 7.71–7.69 (m, 2H), 7.65–7.63 (m, 2H), 7.53 (dd, *J* = 2.4, 2.4 Hz, 1H), 7.49 (s,1H), 7.46 (t, *J* = 7.8 Hz, 2H), 7.34 (t, *J* = 7.2 Hz, 1H), 7.25 (d, *J* = 9.0 Hz, 1H), 5.56 (s, 2H), 3.86 (s, 3H), 3.65 (s, 3H) and 2.09 (s, 3H); ^13^C NMR (ppm, 151 MHz, DMSO-*d*_6_): δ = 168.9, 164.8, 153.6, 150.7, 147.8, 142.5, 139.6, 139.2, 133.2, 129.1, 128.6, 128.2, 128.2, 127.8, 127.2, 126.4, 124.5, 124.1, 118.3, 113.8, 110.4, 108.2, 67.2, 56.2, 56.1, 24.3; HRMS-ESI (*m*/*z*): [M + H]^+^ calcd for C_30_H_27_N_3_O_7_, 542.1922; found, 542.1926; HPLC: *t*_R_ = 13.66 min (96.6% purity).

## Supplementary Material



## Data Availability

A preprint version of this study was posted on ChemRxiv preprint server.^51^

## Notes

Supplemental figures and tables, NMR spectra (^1^H and ^13^C), and HPLC data of tested compounds (PDF)

## Abbreviations

AMC: 7-amino-4-methylcumarin
DMD: *Duchenne* muscular dystrophy
DMNB: 4,5-dimethoxy-2-nitrobenzyl
DMSO: dimethyl sulfoxide
E*I: stable enzyme inhibitor complex
EC_50_: half maximal effective concentration
EI: enzyme inhibitor complex
FDA: Food and Drug Administration
FPU: foot pocket unit
HAT: histone acetyltransferase
HDAC: histone deacetylase
HPLC: high-performance liquid chromatography
HSP90: heat shock protein 90
IC_50_: half maximal inhibitory concentration
*k*_obs_: observed rate constant
MDA-MB-231: M D Anderson metastatic breast 231
MTT: 3-(4,5-dimethylthiazol-2-yl)-2,5-diphenyltetrazolium bromide
NAD^+^: nicotinamide adenine dinucleotide
PI: propidium iodide
PPG: photoremovable protecting groups
SD: standard deviation
SIRT: sirtuin
STAT3: signal transducer and activator of transcription 3
UV: ultraviolet
vor.: vorinostat
ZBG: zinc binding group
