# Supplementary material for: Ortho-Hydroxyanilides: Slow-Acting, Selective Histone Deacetylase 1/2 Inhibitors Suitable for Photocaging Applications
Source: ACS Pharmacol Transl Sci. 2025 Nov 14;8(12):4385–98. doi: 10.1021/acsptsci.5c00562 (PMC12706802; doi:10.1021/acsptsci.5c00562)
Supplement: Supplementary file 1 [file pt5c00562_si_001.pdf]

Supplementary Information

Ortho-Hydroxyanilides: Slow-Acting, Selective Histone Deacetylase 1/2 Inhibitors Suitable for Photocaging Applications

*Irina Honin,[a] Tao Sun,[a], Nisha Setia,[a] Linda Schäker-Hübner,[a] Finn K. Hansen[a]**

[a]Department of Pharmaceutical and Cell Biological Chemistry, Pharmaceutical Institute, University of Bonn, An der Immenburg 4, 53121 Bonn, Germany.

* Correspondence to: finn.hansen@uni-bonn.de

Prof. Dr. Finn K. Hansen, Pharmaceutical and Cell Biological Chemistry, Pharmaceutical Institute, University of Bonn, An der Immenburg 4, 53121 Bonn, Germany. Tel.: (+49) 228 73 5213. Fax: (+49) 228 73 7929. E-mail: finn.hansen@uni-bonn.de.

This supplementary information consists of 10 pages, including 5 Supplementary Figures, and 1 Supplementary Table

TABLE OF CONTENTS

1. SUPPLEMENTARY FIGURES S-3
2. NMR DATA OF SYNTHESIZED COMPOUNDS S-6
3. HPLC CHROMATOGRAMS S-9

1. SUPPLEMENTARY FIGURES

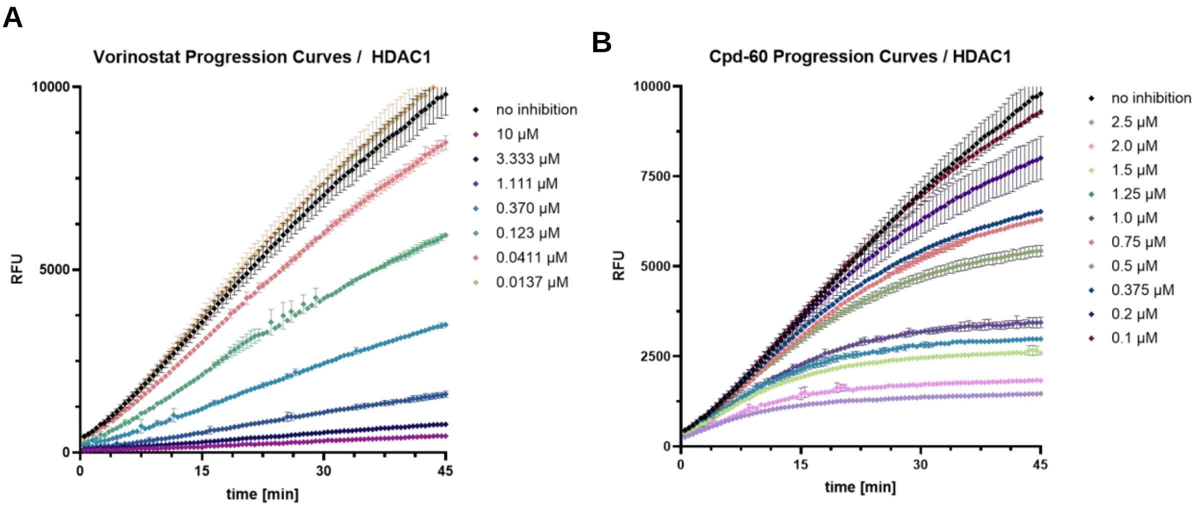

Figure S1. Typical progression curves for the inhibition of HDAC1 by different concentration of a fast-on / fast-off inhibitor like vorinostat (**A**) and slow-binding inhibitor like Cpd-60 (**B**). Representative curves are shown. Data represent mean ± SD, experiments were performed at least twice.

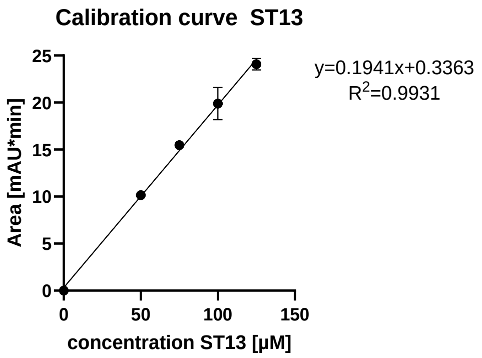

Figure S2. Calibration curve of **ST13**.

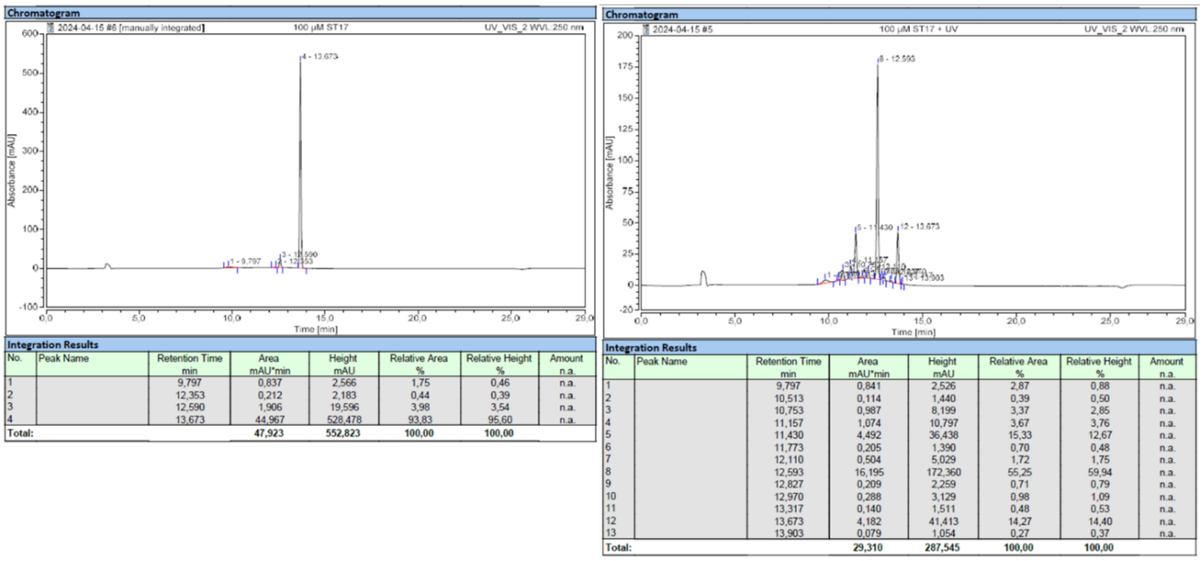

Integration Results

No.	Peak Name	Retention Time min	Area mAU*min	Height mAU	Relative Area %	Relative Height %	Amount n.a.
1		9,797	0,837	2,566	1,75	0,46	n.a.
2		12,353	0,212	2,183	0,44	0,39	n.a.
3		12,590	1,906	19,596	3,98	3,54	n.a.
4		13,673	44,967	528,478	93,83	95,60	n.a.
Total:			47,923	552,823	100,00	100,00	

Integration Results

No.	Peak Name	Retention Time min	Area mAU*min	Height mAU	Relative Area %	Relative Height %	Amount n.a.
1		9,797	0,841	2,526	2,87	0,88	n.a.
2		10,513	0,114	1,440	0,39	0,50	n.a.
3		10,753	0,987	8,199	3,37	2,85	n.a.
4		11,157	1,074	10,797	3,67	3,76	n.a.
5		11,430	4,492	36,438	15,33	12,67	n.a.
6		11,773	0,205	1,390	0,70	0,48	n.a.
7		12,110	0,504	5,029	1,72	1,75	n.a.
8		12,593	16,195	172,360	55,25	59,94	n.a.
9		12,827	0,209	2,259	0,71	0,79	n.a.
10		12,970	0,288	3,129	0,98	1,09	n.a.
11		13,317	0,140	1,511	0,48	0,53	n.a.
12		13,673	4,182	41,413	14,27	14,40	n.a.
13		13,903	0,079	1,054	0,27	0,37	n.a.
Total:			29,310	287,545	100,00	100,00	

Figure S3. Chromatograms of **ST17** prior to irradiation (left) and after 10 minutes of irradiation (right) with light of 365 nm wavelength. The initial **ST17** concentration is 100 µM, while the concentration of released **ST13** (t_R = 12.593 min) following irradiation is 81.67 µM.

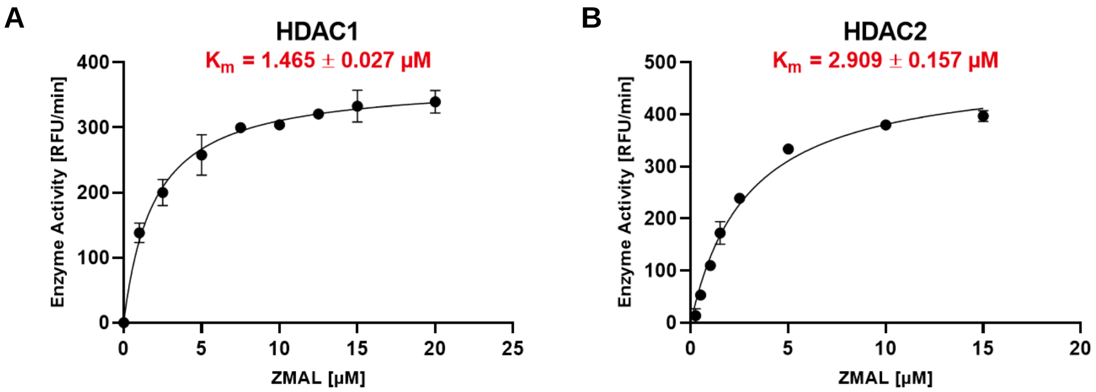

Figure S4. K_m determination for HDAC1 (**A**) and HDAC2 (**B**). Steady-state velocities were plotted against the corresponding substrate concentration and were fitted to the Michaelis-Menten equation. Data represent mean ± SD, experiments were performed at least twice.

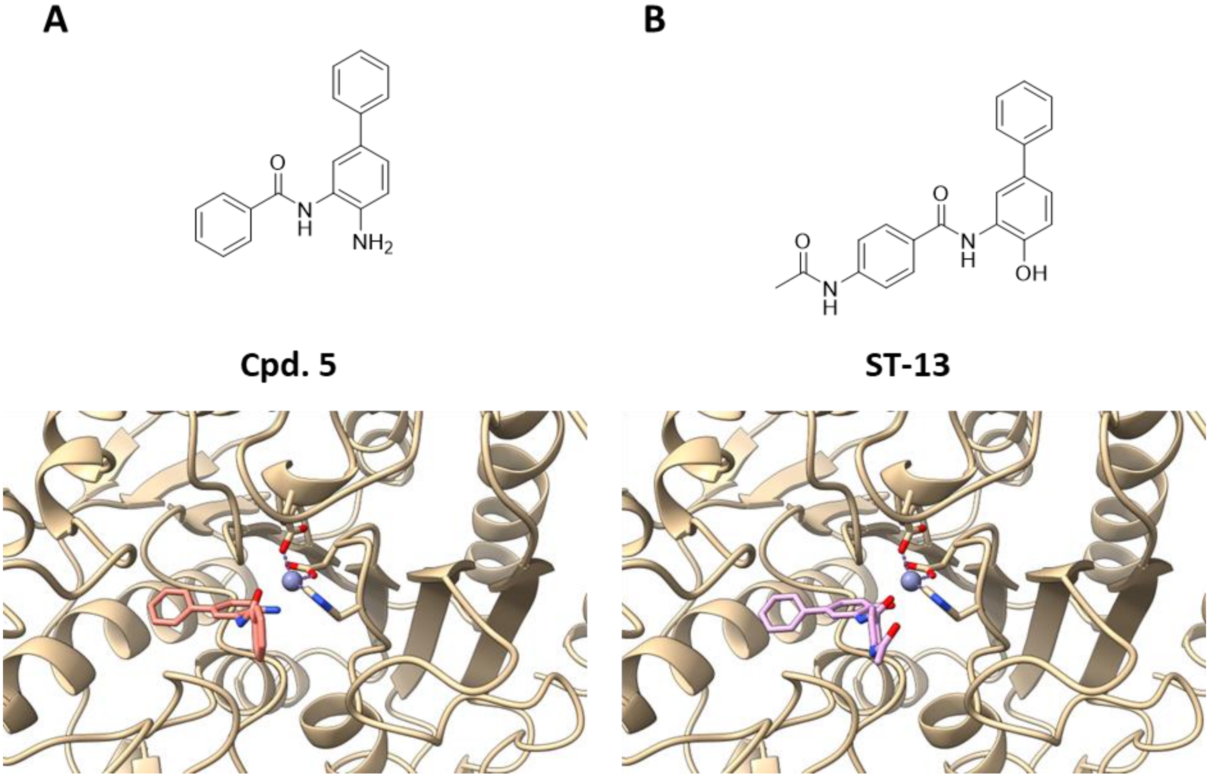

Figure S5. Docking studies. **(A)** Crystal structure of Cpd. **5** in complex with HDAC2 (PDB ID: 3MAX). **(B)** Docking pose of **ST-13** in HDAC2. The catalytic Zn^{2+} ion is shown as a gray sphere.

Table S1. Antiproliferative activity (CellTiter-Glo® cell viability assay) of **ST13,** Cpd-60, and vorinostat against the HEK293 and MM.1S cell lines.

	**EC_{50} [µM]** [a,b]	
	**HEK293**	**MM.1S**
**ST13**	> 5.00 [c]	0.198 ± 0.096
Cpd-60	1.50 ± 1.28	0.111 ± 0.038
vorinostat	0.636 ± 0.226	0.388 ± 0.031

[a] EC_{50} values are reported as mean ± SD from at least two independent experiments. [b] 120 h pre-incubation of cells and inhibitor. [c] < 25% inhibition at the stated concentration.

2. NMR DATA OF SYNTHESIZED COMPOUNDS

^{1}H NMR spectrum of **ST01** (600 MHz, DMSO-d_6)

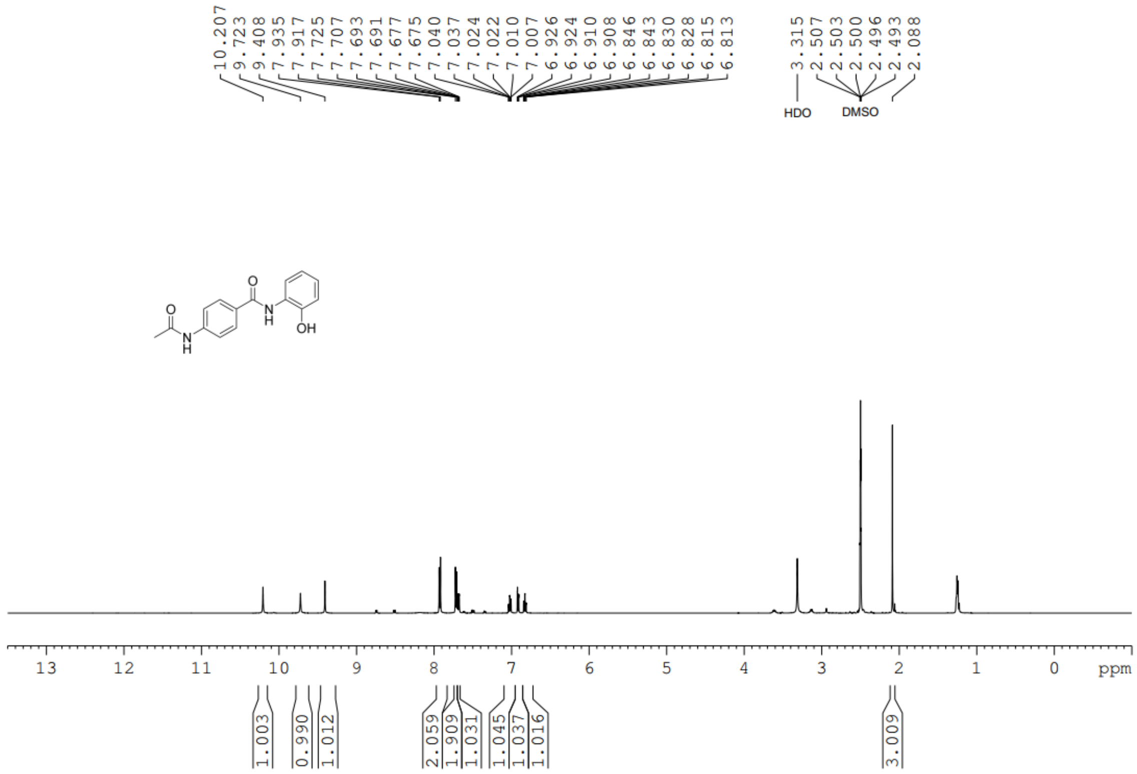

^{13}C NMR spectrum of **ST01** (126 MHz, DMSO-d_6)

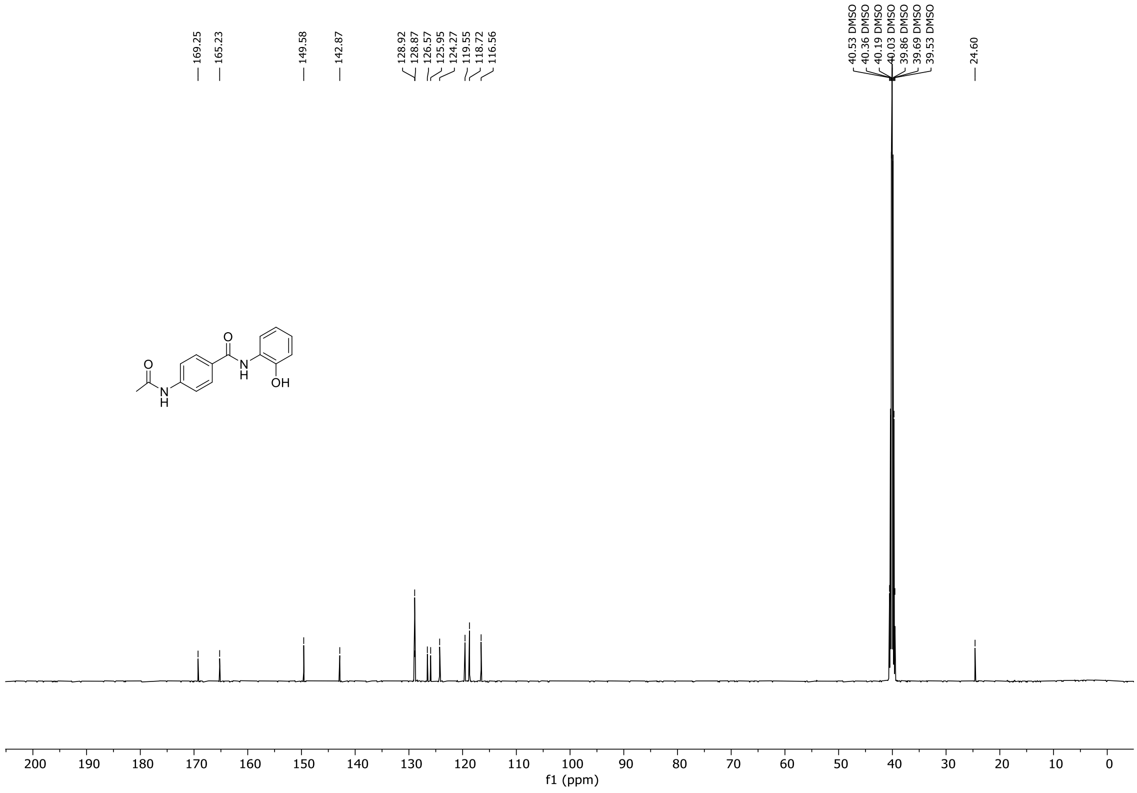

^{1}H NMR spectrum of **ST13** (500 MHz, DMSO-d_6)

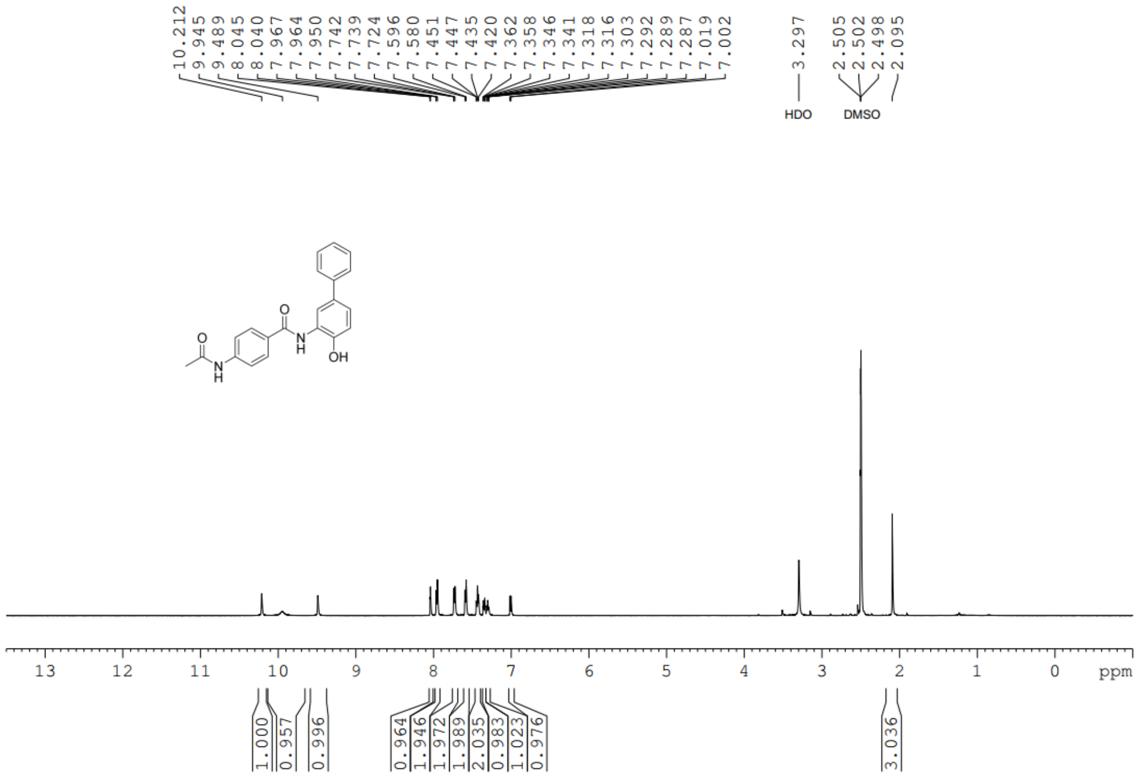

^{13}C NMR spectrum of **ST13** (151 MHz, DMSO-d_6)

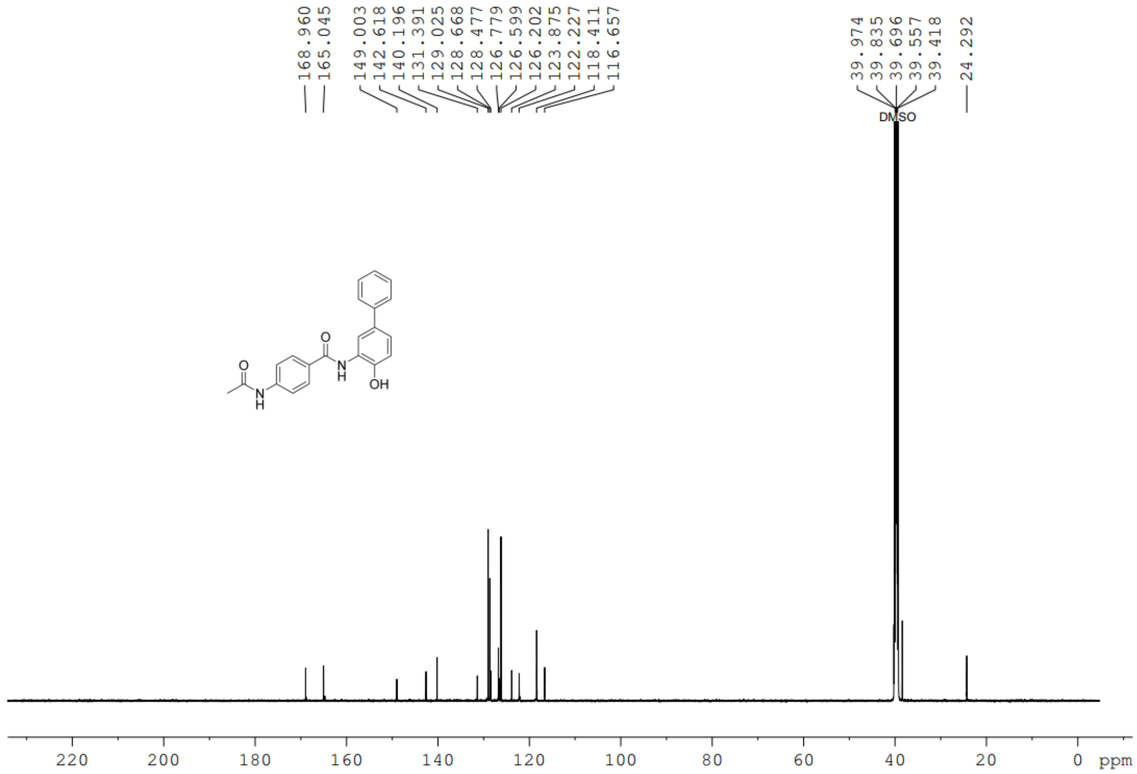

^{1}H NMR spectrum of **ST17** (600 MHz, DMSO-d_6)

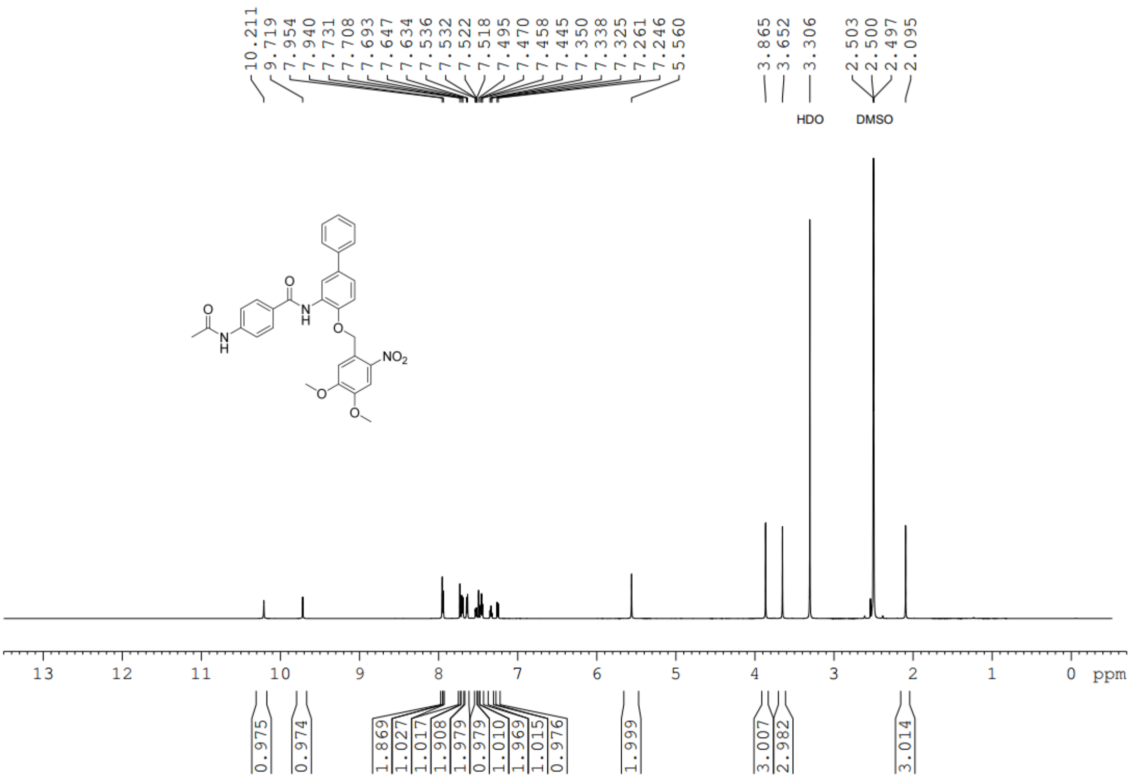

^{13}C NMR spectrum of **ST17** (151 MHz, DMSO-d_6)

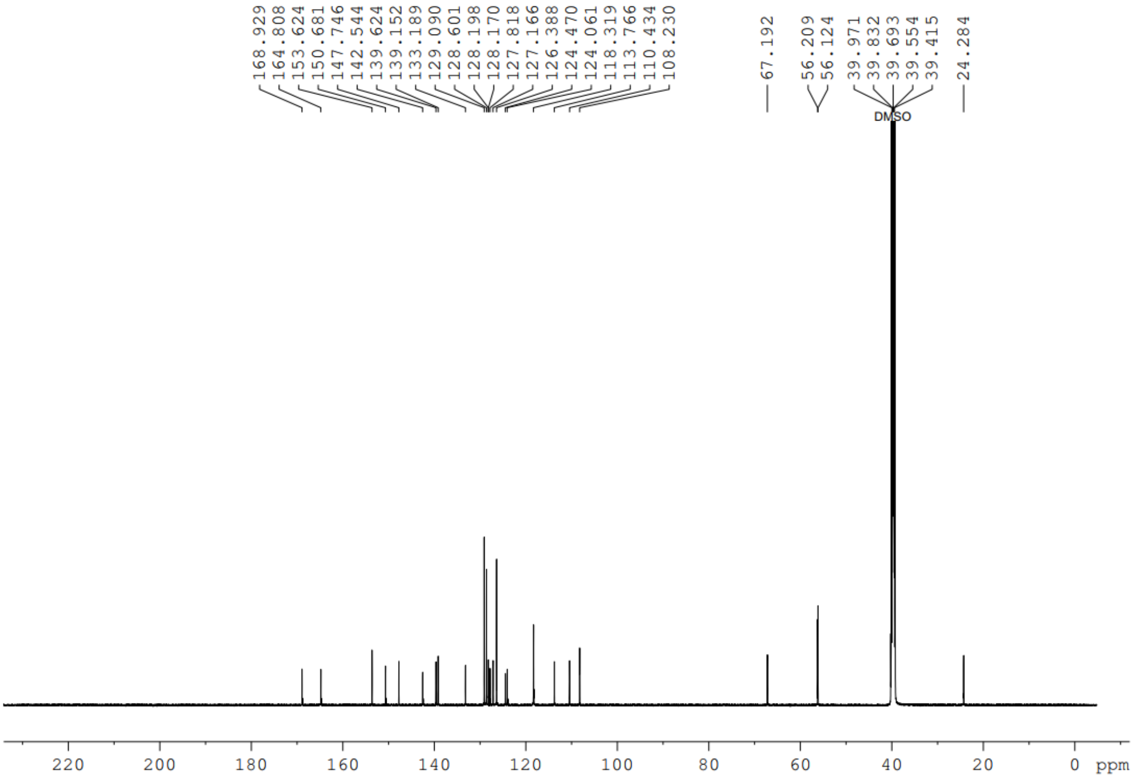

3. HPLC CHROMATOGRAMS

HPLC Chromatogram of **ST01**, purity 96.5%.

Chromatogram

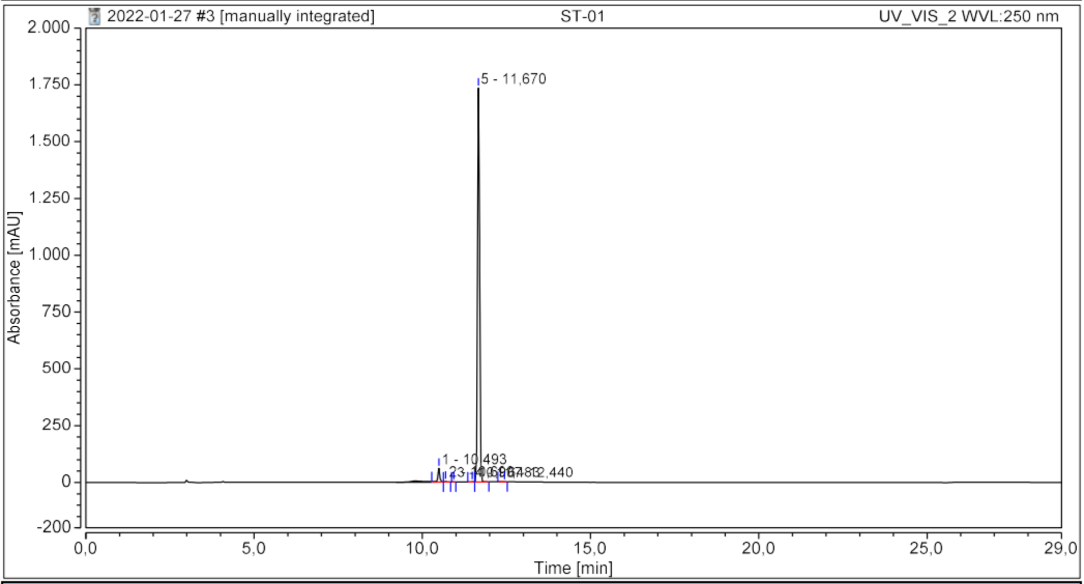

Integration Results

No.	Peak Name	Retention Time min	Area mAU*min	Height mAU	Relative Area %	Relative Height %	Amount n.a.
1		10,493	3,933	60,293	3,14	3,35	n.a.
2		10,690	0,241	3,582	0,19	0,20	n.a.
3		10,937	0,068	1,199	0,05	0,07	n.a.
4		11,483	0,105	1,344	0,08	0,07	n.a.
5		11,670	120,978	1733,985	96,49	96,29	n.a.
6		12,440	0,053	0,386	0,04	0,02	n.a.
**Total:**			**125,378**	**1800,789**	**100,00**	**100,00**	

HPLC Chromatogram of **ST13**, purity 96.9%.

Chromatogram

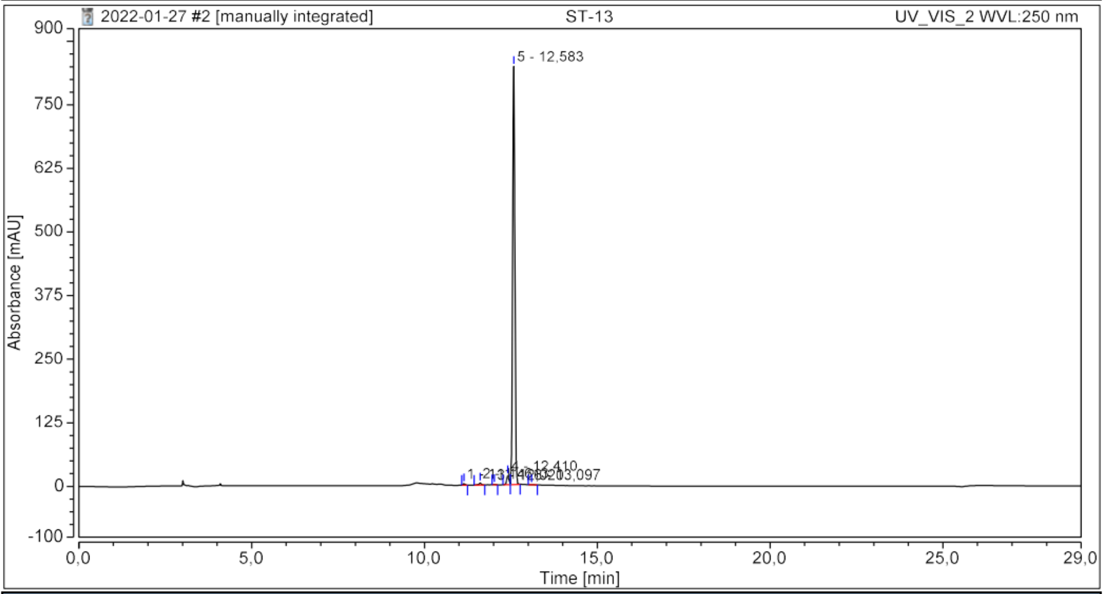

Integration Results

No.	Peak Name	Retention Time min	Area mAU*min	Height mAU	Relative Area %	Relative Height %	Amount n.a.
1		11,147	0,197	3,115	0,32	0,37	n.a.
2		11,613	0,346	4,290	0,56	0,51	n.a.
3		12,020	0,060	0,886	0,10	0,10	n.a.
4		12,410	1,254	17,839	2,04	2,10	n.a.
5		12,583	59,465	822,275	96,89	96,88	n.a.
6		13,097	0,052	0,388	0,08	0,05	n.a.
**Total:**			**61,375**	**848,793**	**100,00**	**100,00**	

HPLC Chromatogram of **ST17**, purity 96.6%.

Chromatogram

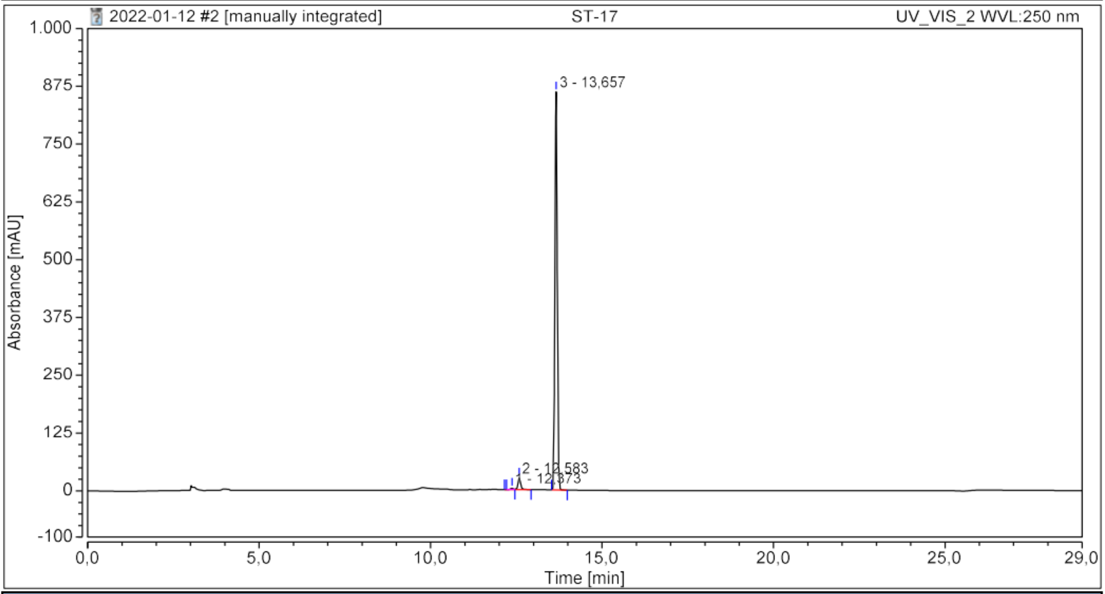

Integration Results

No.	Peak Name	Retention Time min	Area mAU*min	Height mAU	Relative Area %	Relative Height %	Amount n.a.
1		12,373	0,239	3,161	0,34	0,36	n.a.
2		12,583	2,192	25,165	3,09	2,83	n.a.
3		13,657	68,511	861,088	96,57	96,82	n.a.
**Total:**			**70,942**	**889,414**	**100,00**	**100,00**	
